# Clinical relevance and therapeutic predictive ability of hypoxia biomarkers in head and neck cancer tumour models

**DOI:** 10.1002/1878-0261.13620

**Published:** 2024-03-01

**Authors:** Tet Woo Lee, Dean C. Singleton, Julia K. Harms, Man Lu, Sarah P. McManaway, Amy Lai, Moana Tercel, Frederik B. Pruijn, Andrew M. J. Macann, Francis W. Hunter, William R. Wilson, Stephen M. F. Jamieson

**Affiliations:** ^1^ Auckland Cancer Society Research Centre University of Auckland New Zealand; ^2^ Maurice Wilkins Centre for Molecular Biodiscovery University of Auckland New Zealand; ^3^ Department of Molecular Medicine and Pathology University of Auckland New Zealand; ^4^ Department of Pharmacology and Clinical Pharmacology University of Auckland New Zealand; ^5^ Department of Radiation Oncology Auckland City Hospital New Zealand; ^6^ Oncology Therapeutic Area Janssen Research and Development Spring House PA USA

**Keywords:** evofosfamide, head and neck squamous cell carcinoma, hypoxia, hypoxia‐activated prodrugs, pimonidazole, tumour xenografts

## Abstract

Tumour hypoxia promotes poor patient outcomes, with particularly strong evidence for head and neck squamous cell carcinoma (HNSCC). To effectively target hypoxia, therapies require selection biomarkers and preclinical models that can accurately model tumour hypoxia. We established 20 patient‐derived xenograft (PDX) and cell line‐derived xenograft (CDX) models of HNSCC that we characterised for their fidelity to represent clinical HNSCC in gene expression, hypoxia status and proliferation and that were evaluated for their sensitivity to hypoxia‐activated prodrugs (HAPs). PDX models showed greater fidelity in gene expression to clinical HNSCC than cell lines, as did CDX models relative to their paired cell lines. PDX models were significantly more hypoxic than CDX models, as assessed by hypoxia gene signatures and pimonidazole immunohistochemistry, and showed similar hypoxia gene expression to clinical HNSCC tumours. Hypoxia or proliferation status alone could not determine HAP sensitivity across our 20 HNSCC and two non‐HNSCC tumour models by either tumour growth inhibition or killing of hypoxia cells in an *ex vivo* clonogenic assay. In summary, our tumour models provide clinically relevant HNSCC models that are suitable for evaluating hypoxia‐targeting therapies; however, additional biomarkers to hypoxia are required to accurately predict drug sensitivity.

## Graphical abstract

Tumour hypoxia promotes poor prognosis in head and neck squamous cell carcinoma (HNSCC). We established 20 patient‐derived xenograft (PDX) and cell line‐derived xenograft (CDX) models of HNSCC. PDX models were significantly more hypoxic than CDX models and showed similar hypoxia gene expression to clinical HNSCC tumours. Hypoxia status alone could not predict sensitivity to hypoxia‐activated prodrugs across our tumour models.

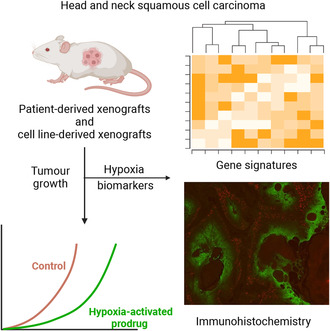

**Abbreviations**
CCLE: Cancer Cell Line Encyclopaedia
CCN: CancerCellNet
CDX: cell line‐derived xenograft
EdU: 5‐ethynyl‐2′‐deoxyuridine
FCS: foetal calf serum
GDC: Genomic Data Commons
HAPs: hypoxia‐activated prodrugs
HNSCC: head and neck squamous cell carcinoma
HPV: human papilloma virus
nitroCBIs: nitrochloromethylbenzindolines
PDX: patient‐derived xenograft
RLE: relative log expression
SCC: squamous cell carcinoma
STR: single tandem repeat
TCGA: the Cancer Genome Atlas

## 1 Introduction

Head and neck cancers in the oral and nasal cavities, pharynx, larynx and salivary glands collectively constitute the seventh most common cancer diagnosis and cause of cancer‐related mortality [1]. Approximately 90% of head and neck cancers originate in the squamous epithelial cells lining the mucosal surface of these tissues and are termed head and neck squamous cell carcinoma (HNSCC) [2]. Human papillomavirus (HPV) infection is responsible for causing approximately 20–30% of HNSCC, which are more responsive to therapy than HPV‐negative disease [3, 4, 5].

A major negative prognostic factor for HPV‐negative HNSCC is tumour hypoxia [6, 7, 8], which is more prevalent in HNSCC than other tumour types [9]. Areas of low oxygen within the tumour microenvironment can promote resistance to chemotherapy, radiotherapy and immunotherapy [10] and therefore influence patient outcome. Various treatment strategies have been designed to target hypoxia and provide tumour selectivity due to the lack of severe hypoxia in normal tissues [7, 10]. One such strategy is the use of hypoxia‐activated prodrugs (HAPs) that undergo tumour‐selective metabolism to the active drug by one‐electron reductases under low oxygen [11]. Several HAPs have been developed, including tirapazamine, PR‐104 and evofosfamide [12, 13, 14], which all entered clinical development, but failed, likely due at least in part to the absence of prospective biomarkers to preselect suitable patients [15]. Continued evaluation of emerging classes of HAPs is ongoing, including the irreversible EGFR inhibitor prodrug tarloxotinib [16], the nitrogen mustard prodrug CP‐506 [17, 18] and nitrochloromethylbenzindolines (nitroCBIs) [19, 20], with the inclusion of selection biomarkers necessary for their future success as are preclinical models that can accurately model the pathophysiology of tumour hypoxia.

As HAPs require hypoxia for activation, measures that can differentiate more hypoxic tumours from less hypoxic tumours are prime candidates for HAP patient stratification. Other potential biomarkers include genetic markers of sensitivity to the released effector, e.g., proliferation markers [13], DNA repair defects [18], and genes encoding HAP reductases such as AKR1C3 for aerobic activation of PR‐104 [21] and STEAP4 for hypoxic activation of tarloxotinib [22]. While imaging modalities, such as positron emission tomography and oxygen‐enhanced MRI, are preferred methods for detecting tumour hypoxia in the clinic [23, 24], very few laboratories have this capability for preclinical studies. Immunohistochemical detection of covalently bound metabolites of pimonidazole or EF5 has been the most common hypoxia marker in preclinical models [25, 26]. These 2‐nitroimidazoles offer the potential advantage of reporting both hypoxia and one‐electron reductase activity in hypoxia cells [27], but do not allow repeat measurements in the same tumour over time unless multiple markers are used [28, 29, 30]. More recently, a number of different gene signatures have been developed that can differentiate highly hypoxic cells or tumours from those that are less hypoxic, including several that were developed specifically from HNSCC samples [31, 32, 33, 34, 35, 36]. Additionally, the Buffa gene signature that was developed from patients across multiple cancer types [37] has been used to show that HNSCC tumours are more hypoxic than other tumour types [9]. A separate 15 gene signature was developed by Toustrup et al. using HNSCC tumour biopsies [35] that identified more hypoxic tumours and could retrospectively predict response to the hypoxia‐dependent radiosensitiser nimorazole [38]. Unfortunately, a prospective follow‐up trial was terminated after only 194 of the planned 640 patients had been recruited [39], so further research is required to evaluate whether hypoxia gene signatures have utility in this setting.

Many tumour models of HNSCC exist, but their ability to accurately model clinical tumour hypoxia is unclear as higher hypoxic fractions have been reported in cell line xenograft and patient‐derived xenograft models than patient tumours [7]. We have previously evaluated hypoxic fractions for a small number of HNSCC patient‐derived xenograft (PDX) models by pimonidazole immunohistochemistry, which we found were in a similar range to published clinical data [40]. Here, we extend our collection of HNSCC models with additional PDX models and cell line‐derived xenograft (CDX) models and evaluate these, along with SiHa and an HCT116 subline as two other well‐studied CDX models, for hypoxia and proliferation status using histochemical markers and gene signatures. We also assess the utility of these potential biomarkers for hypoxia targeting with HAPs by either *in vivo* tumour growth inhibition or *ex vivo* clonogenic assay following treatment.

## 2 Materials and methods

### 2.1 Cell culture

UT‐SCC‐1A (RRID:CVCL_7863), UT‐SCC‐16A (RRID:CVCL_7812), UT‐SCC‐42B (RRID:CVCL_7848), UT‐SCC‐74A (RRID:CVCL_A7DX), UT‐SCC‐74B (RRID:CVCL_A7DY), UT‐SCC‐76A (RRID:CVCL_A7DZ), UT‐SCC‐110B (RRID:CVCL_A091), UT‐SCC‐126A (RRID:CVCL_A7EC) were derived by Prof. Reidar Grénman (University of Turku, Finland), supplied by Prof. Bradly Wouters (University Health Network, Toronto) along with HCT116/54C cells (see below) and were cultured in minimum essential media (MEM, ThermoFisher Scientific, Auckland, New Zealand) with 10% foetal calf serum (FCS, Moregate Biotech, Hamilton, New Zealand), 4.5 mg·mL^−1^ D‐glucose (Sigma‐Aldrich, Auckland, New Zealand) and 20 mM HEPES (Sigma‐Aldrich). SiHa (RRID:CVCL_0032; supplied by Dr David Cowan, Ontario Cancer Institute, Toronto), FaDu (RRID:CVCL_1218; American Type Culture Collection, Manassas, VA) were cultured in αMEM (ThermoFisher Scientific) with 5% and 10% FCS, respectively. All cultures were passaged without antibiotics, were confirmed to be Mycoplasma negative by PlasmoTest (InvivoGen, San Diego, CA, USA) and were derived from single tandem repeat (STR)‐authenticated frozen stocks.

### 2.2 Animals

All animal experiments were approved by the University of Auckland Animal Ethics Committee (approvals: #001781 and #002256). NOD scid gamma mice (NOD.Cg‐*Prkdc* ^*scid*^ *Il2rγ* ^*tm1Wjl*^ */SzJ*) were supplied by Jackson Laboratory (Bar Harbour, ME, USA) and NOD scid (NOD.CB17‐*Prkdc* ^*scid*^/NCrCrl) and NIH‐III mice (NIH‐*Lyst* ^*bg‐J*^ *Foxn1* ^*nu*^ *Btk* ^*xid*^) by Charles River Laboratories (Wilmington, MA, USA), and were bred in the Vernon Jansen Unit, University of Auckland. Animals had *ad libitum* access to food and water in microisolator cages and were maintained on a 12 h light/dark cycle. Animal health and welfare was monitored regularly with animals euthanised if their condition deteriorated or if their pre‐manipulation bodyweight declined by 20%. Tumour volume was recorded by electronic callipers using the formula π/6 × width × length^2^.

### 2.3 Patient‐derived xenograft (PDX) and cell line‐derived xenograft (CDX) models

PDX models were established from HNSCC patient tumour specimens collected at Auckland City Hospital between December 2014 and March 2021. The study methodologies conformed to the standards set by the Declaration of Helsinki, were undertaken with the understanding and written informed consent of each subject, and were approved by the New Zealand Health and Disability Ethics Committee (approval: 14/NTB/122) and the Auckland District Health Board. Small tumour fragments were engrafted into 6‐ to 8‐week‐old female NOD scid gamma or NOD scid mice as described previously [40]. Tumours were collected and dissected into ~ 1 mm^3^ fragments for engraftment into additional mice as they reached approximately 1500 mm^3^. Third‐generation (P3) PDX tumours were generated by subcutaneous bilateral engraftment of three P2 tumour fragments into NOD scid mice. CDX models were established by inoculating 6‐ to 8‐week‐old female NIH‐III mice with 5 × 10^6^ cells subcutaneously onto one or both flanks. Once CDX or P3 PDX tumours reached ~ 250 mm^3^, animals were either euthanised for tumour collection immediately or 2 h following IP treatment with 60 mg·kg^−1^ pimonidazole (NPI Inc., Burlington, MA, USA) and/or 50 mg·kg^−1^ EdU (5‐ethynyl‐2′‐deoxyuridine, Abcam, Cambridge, UK), or were recruited for HAP treatment.

### 2.4 HAP treatment of mice

To assess the antitumour efficacy of evofosfamide, recruited animals were treated with 50 mg·kg^−1^ evofosfamide (Threshold Pharmaceuticals Inc., San Francisco, CA, USA and Medkoo Biosciences, Morrisville, NC, USA) in saline or control vehicle (saline) by IP injection at qdx5 for 3 weeks. Tumours were measured two to three times a week by electronic callipers until they reached endpoint (~ 2000 mm^3^). To estimate tumour growth rates, a Gompertz curve was fitted to the growth measurement data for each tumour as a data smoothing function. To fit these curves, the initial tumour volume at treatment onset (W_0_) was first estimated by linear regression of all data points from 7 days prior to treatment to 5 days after commencing treatment (inclusive). U‐Gompertz functions (W_0_‐forms with absolute growth rate K_u_ and asymptote A parameters) [41] were then fitted to the tumour volume (*W*) for each measurement time point (*t*; with 0 as treatment start day) using non‐linear least squares using nlsLM function from the *minpack.lm* package in *R* with *W*_0_ fixed to the previously estimated starting volume. The estimated fit parameters were not analysed directly but rather the fitted curves were used to provide smooth functions of tumour volume over time despite measurement noise; all fitted curves were inspected to ensure correspondence with the raw data. The mean daily tumour growth rate (mm^3^·day^−1^) for each tumour across the treatment period was then estimated by taking the mean of the slope of the fitted curve at each day (i.e. taking the derivative at *t* = 1, 2, … 21 days using finite difference). Where tumours decreased in size, daily growth estimation excluded days when tumour size was < 10 mm^3^.

For *ex vivo* clonogenic assay, recruited animals were treated with drug as single agents or 5 min after 13 Gy irradiation. Treatments included 150 mg·kg^−1^ evofosfamide (Threshold Pharmaceuticals Inc.) by IP injection in saline, 578 mg·kg^−1^ PR‐104 by IP injection in water‐for‐injection and 12 mg·kg^−1^ (with irradiation) or 40 mg·kg^−1^ (without irradiation) of nitroCBIs SN30726 or SN38737 by IV injection in PBS with four equivalents NaHCO_3_ (all synthesised at the University of Auckland). Control animals were treated with sterile saline by IP injection or with 13 Gy gamma radiation. Irradiation was administered whole body to unrestrained, unanaesthetised mice using a ^60^Co Eldorado 78 radiotherapy unit at a dose rate of 1.7 Gy·min^−1^. Animals were euthanised 18 h following treatment and tumours were excised for dissociation and clonogenic assay as previously described [42].

### 2.5 RNAseq

CDX and PDX tumours were collected at approximately 250 mm^3^ in size, and frozen rapidly in liquid nitrogen. Tissue was homogenised in PureZol (Bio‐Rad Laboratories Inc., Auckland, New Zealand) using a TissueLyser II (Qiagen, Auckland, New Zealand). RNA was extracted from up to 100 mg of pulverised tissue using the Aurum Total RNA Fatty and Fibrous Tissue Kit (Bio‐Rad), with RNA sample quality assessed by Nanodrop ND‐1000 (ThermoFisher Scientific) and quantitated by Qubit BR RNA kit on a Qubit 3.0 fluorometer (ThermoFisher Scientific). Sequencing libraries were prepared using the NEXTFLEX Poly(A) kit (PerkinElmer, Waltham, MA, USA) for poly(A) enrichment and NEXTFLEX Rapid Directional qRNA‐seq Kit (PerkinElmer) or NEXTFLEX Rapid Directional qRNA‐Seq Kit 2.0 (PerkinElmer) for cDNA library preparation. Library integrity was confirmed on a TapeStation 2200 (Agilent Technologies, Santa Clara, CA, USA) and sequenced on a NextSeq500 (Illumina, San Diego, CA, USA) using high‐output flow cells (Illumina) with 150 bp paired ends.

Reads were analysed using a Snakemake‐based pipeline available at https://gitlab.com/twlee79/hnscc_clxpdx. Reads were trimmed using *trim_galore* to remove adaptors, very low‐quality reads (minimal Phred score of 10 [43]) and reads < 50 bp in length. Trimmed reads were aligned to the hg38 and mm10 genomes (primary assemblies from Gencode v37 and Gencode vM25) using STAR and disambiguated to human and mouse reads using *disambiguate*. When unique molecular labels were present (qRNA‐seq Kit), these were extracted to the read names using *UMI‐tools* prior to the *disambiguate* step. Disambiguated reads were realigned to the respective genomes and the realigned reads were deduplicated using *UMI‐tools*. For better comparisons to TCGA RNASeq data, the disambiguated/deduplicated human reads from each sample were then run through the Genomic Data Commons (GDC) Dr32 pipeline (https://docs.gdc.cancer.gov/Data/Bioinformatics_Pipelines/Expression_mRNA_Pipeline/) using the specified parameters and genome indexes. The implementation of this pipeline used is available at https://gitlab.com/twlee79/hnscc_clxpdx_gdc. Cell line RNASeq data were obtained from the NCBI SRA under accession numbers (PRJNA477597 and PRJNA523380) and processed through the same pipeline, including alignment to the mm10 genome and *disambiguate*, to ensure identical processing of the data. TCGA GDC RNASeq count data (Data Release 37.0) was directly downloaded from the GDC Data Portal.

Viral reads were determined using a version of the VirTect pipeline [44] implemented in https://gitlab.com/twlee79/hnscc_clxpdx. Unmapped hg38 reads from the initial alignment were aligned using BWA against the VirTect 757 virus database. HPV status was defined to be positive when there were at least 400 reads mapping to an HPV genome and these were aligned to a continuous region of at least 200 bp of that genome at a minimum depth of 5.

### 2.6 Cancer CellNet analysis

CancerCellNet (CCN) analysis [45] was conducted with the *cancerCellNet* package in R using RNASeq data from the GDC Release 37.0 data. The annotations and parameters for a general classifier provided by the original authors were used. For comparisons to Cancer Cell Line Encyclopaedia (CCLE) cell lines, CCLE 2019 RNASeq counts [46] were obtained from the DepMap portal. The genes used when training the classifiers were limited to genes in common between the GDC (Gencode v36) and CCLE pipeline (Gencode v19), matched by Ensembl Gene ID (23 335 genes after filtering by *cancerCellNet*). Once trained with the TCGA data, CCN scores were determined from RNASeq data for each of our cell line and xenograft samples, as well as the CCLE cell lines.

### 2.7 Statistical analysis of RNASeq data

RNASeq data were normalised using the relative log expression (RLE) method and genes with low counts filtered (minimum count of 10 in at least one sample, and minimum total count of 15) with counts of 21 572 genes remaining. Data were analysed using *dream* [47] to fit a linear mixed model with ‘model’ (cell line or PDX model number) as a random effect and sample type (cultured cells, CDX or PDX) as a fixed effect; this model will conduct a paired comparison between CDX and the corresponding cells, and unpaired comparisons for PDX. The statistical comparison conducted were an F‐test for difference across sample types, and individual contrasts for comparisons between CDX and cells, PDX and cells, as well as PDX vs CDX. Gene set tests were conducted with the *zenith* package, which implements the *camera* method [48] for *dream* mixed models, using the 50 MSigDB Hallmark gene sets [49], and inter‐gene correlation set at the *zenith* default of 0.01. These tests were ‘competitive’ with the null hypothesis for the gene set tests being that the average log fold‐change of the genes in the gene set was the same as the average log fold‐change for genes not in the set [48].

### 2.8 Hypoxia gene signatures

RNASeq read counts were combined with the TCGA‐HNSCC RNASeq counts from the GDC and normalised using the RLE method. Only TCGA Primary Tumours (with HPV status assigned as previously reported [50]) and TCGA Normal samples were included. Hypoxia signature scoring was determined by first generating a z‐score for each gene in each sample relative to the tumour xenograft samples only, where some hypoxia was expected to be present (i.e., the mean normalised read count for that sample minus the mean expression across all tumour xenograft samples and divided by the standard deviation across tumour xenograft samples). All *z*‐scores for individual genes were constrained to values of between −3 and 3. The *z*‐scores for each gene were summed across all genes in the signature to give a signature score. To allow comparison across hypoxia gene signatures that had different numbers of genes and ranges of expression, a simple signature score was first determined, as previously described [9], giving a score of +1 for every gene in that sample that was above the median mRNA normalised count for that gene across all the xenograft samples and a score of −1 for every gene that was below the median mRNA normalised count, with the maximum possible score being the total number of genes in that signature. Each signature score was normalised to the number of genes present in the signature to give a Hypoxia Score ranging between 0 and 1.

### 2.9 Histochemistry

Tumour samples were fixed, sectioned, mounted onto slides and imaged for pimonidazole (470 nm), EdU (630 nm) and Hoechst 33258 (365 nm) as described previously [40].

### 2.10 Statistics

Differences in means between two groups were evaluated by paired or unpaired Student's *T*‐test and between more than two groups by one‐way ANOVA with Sidak's or Dunnett's multiple comparison analysis. Pearson correlation coefficients were determined for correlation analyses. All statistical analyses were carried out using prism v9.3.1 (GraphPad, San Diego, CA, USA).

## 3 Results

### 3.1 Tumour xenograft models of HNSCC

**Fig. 1:**
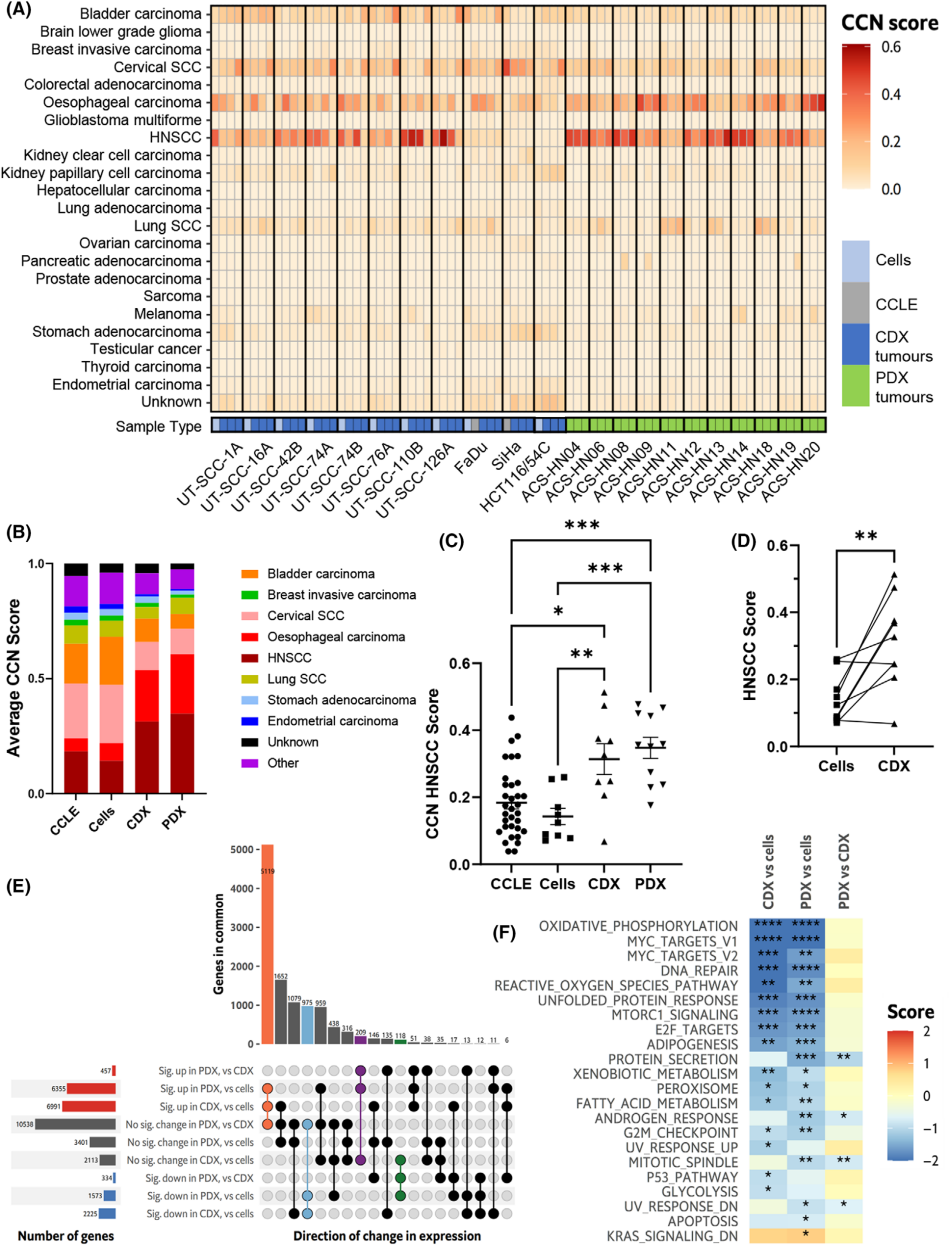
Transcriptional fidelity of head and neck squamous cell carcinoma (HNSCC) tumour models. (A) CancerCellNet (CCN) scores for 22 tumour models and cells across different cancer types. (B) Average CCN Score and (C) CCN HNSCC Score for cancer cell line encyclopaedia HNSCC cell lines (CCLE, *n* = 33), our cancer cell lines (Cells, *n* = 9), HNSCC cell line‐derived xenograft (CDX, *n* = 9) and patient‐derived xenograft (PDX, *n* = 11) tumours. (D) CCN HNSCC Score for CDX tumours and their paired cell line (Cells; *n* = 9). (E) UpSet plot of genes significantly changed between sample types (cells, CDX and PDX tumours; *F*‐test FDR < 0.05; *n* = 11 329 genes), divided into direction of significant change in each direction of individual contrasts (FDR < 0.05 and absolute log_2_ fold‐change ≥ 1). Intersections of particular interest have been highlighted. Sig = significant. (F) Summary of gene set analysis for comparisons between cells, CDX and PDX tumours with the MsigDB Hallmark gene sets. Score is the mean *t*‐statistic for genes in the set compared to genes not in the set; gene sets where at least one comparison was significant are shown. Line and error bars in (C) and (D) represent mean ± SEM. *P < 0.05; **P < 0.01; ***P < 0.001; ****P < 0.0001 by one‐way ANOVA with Sidak's multiple comparison analysis in (C), paired *T*‐test in (D), and zenith FDR in (F).

We established 20 tumour models of HPV‐negative HNSCC including 9 cell line‐derived xenograft models and 11 patient‐derived xenograft models (Table 1). Eight of these PDX models were generated in a previous study [40], while three new models were established here with an overall success rate of PDX engraftment to P3 of 55% across both studies. We also included the cervical carcinoma line SiHa in our studies as a well‐characterised squamous cell carcinoma (SCC) tumour model that contains predictable fraction of chronically hypoxic cells [51, 52], and an HCT116 colorectal carcinoma cell line now known as HCT116/54C [53]. This cell line was initially derived from a UT‐SCC‐54C culture that was contaminated with HCT116 cells and has now been outgrown by and resembles HCT116 (See Supplementary Results, Table S1 and S2). SiHa and HCT116/54C were excluded for analyses specific for HNSCC. To check the fidelity of our HNSCC tumour models, we carried out RNAseq (Table S3) and evaluated their similarity in human gene expression to clinical HNSCC tumours and HNSCC cell lines using the CancerCellNet (CCN) analysis [45]. All HNSCC tumour samples displayed similarity with clinical TCGA HNSCC tumour samples, generating an average CCN HNSCC score of > 0.15, except for FaDu (0.068 ± 0.012; Fig. 1A), with low to moderate CCN scores also obtained for closely related oesophageal carcinoma, cervical SCC and to a lesser extent bladder carcinoma and lung SCC (Fig. 1B). This pattern of HNSCC samples obtaining scores for other tumour types is consistent with CCN scores of HNSCC cell lines from the cancer cell line encyclopaedia (CCLE) dataset [45] and of those used to generate the CDX tumours (Fig. 1A,B). Average CCN HNSCC score was significantly higher for PDX tumours (0.35 ± 0.03) than for CCLE cell lines (0.18 ± 0.02, *P* < 0.0005) and our cell lines (0.14 ± 0.02, *P* < 0.0005; Fig. 1C), reflecting that the greater complexity of PDX tumours more accurately matches clinical HNSCC tumours than 2D cell lines. The HNSCC CDX tumours also had a significantly higher average CCN HNSCC score (0.31 ± 0.05) than HNSCC cell lines (*P* < 0.01 vs our cell lines and *P* < 0.05 vs CCLE lines), and there was an increased CCN HNSCC score for the CDX tumours compared to the corresponding cell line grown *in vitro* (+0.17 ± 0.05; *P* < 0.01; Fig. 1D). Significantly higher CCN oesophageal carcinoma scores and significantly lower CCN cervical SCC and bladder carcinoma scores were also obtained for both CDX and PDX tumours compared to either the CCLE or the CDX cell lines, while lower lung SCC scores were seen for CDX tumours compared to CCLE cell lines (Fig. S1).

**Table 1:** PDX and CDX tumour models.

Tumour model	Primary site	Type
ACS‐HN04	Tongue	Primary
ACS‐HN06	Oropharynx	Recurrent
ACS‐HN08	Tongue	Primary
ACS‐HN09	Tongue	Primary
ACS‐HN11	Tongue	Recurrent
ACS‐HN12	Tongue	Primary
ACS‐HN13	Hypopharynx pyriform sinus	Primary
ACS‐HN14	Buccal mucosa	Primary
ACS‐HN18	Tongue	Primary
ACS‐HN19	Tongue	Primary
ACS‐HN20	Tongue	Primary
UT‐SCC‐1A	Gingiva	Primary
UT‐SCC‐16A	Tongue	Primary
UT‐SCC‐42B	Supraglottic larynx	Metastasis
UT‐SCC‐74A	Tongue	Primary
UT‐SCC‐74B	Tongue	Recurrent
UT‐SCC‐76A	Tongue	Primary
UT‐SCC‐110B	Gingiva, maxillary sinus	Metastasis
UT‐SCC‐126A	Labii inferioris	Primary
FaDu	Hypopharynx	Primary
SiHa	Uterus	Primary
HCT116/54C	See text	

An overall analysis of gene expression in HNSCC cells grown *in vitro*, CDX tumours and PDX tumours revealed significant changes in gene expression in 11 329 genes across sample types (Fig. 1E). A majority (5119) of these genes showed increased gene expression in CDX and PDX tumours compared to cells, with similar expression between CDX and PDX tumours (Fig. 1E; orange), while a much smaller number of genes (975) showed decreased expression in CDX and PDX tumours compared to cells, and similar CDX/PDX expression (Fig. 1E; light blue). In comparison, only 209 genes showed significantly increased and 118 genes significantly decreased expression in PDX tumours vs either cells or CDX tumours (Fig. 1E; purple/green highlights). With a focus on the 80 diagnostic genes used by CCN to derive HNSCC scores, 55 were significantly changed across sample types (Fig. S2). The predominant pattern again was an increase in expression in the xenografts (36 genes significantly increased expression at FDR < 0.05 in CDX and PDX tumours compared to cells compared to 17 genes significantly decreased in expression). We also conducted competitive gene set tests using the MSigDB hallmark gene sets (Fig. 1F). Reduced expression of several hallmark gene sets was detected for CDX or PDX samples compared to cells including those related to oxidative phosphorylation, MYC targets, DNA repair and reactive oxygen species. Despite there being a general increase in gene expression among the tumour xenografts compared to cells, only a single hallmark gene set was detected with higher expression in tumours versus cells (KRAS Signalling Down in PDX tumours).

RNAseq data was also evaluated for the presence of viral RNA to check for HPV infection. While viral RNA was detected in the known HPV‐positive SiHa samples, none of the HNSCC cell or tumour samples were found to have viral RNA present, indicating that they are all likely HPV‐negative (Table S4).

Principal component analysis was carried out on the expression of the top 500 common genes to determine if the individual tumour replicates clustered with each other as well as whether the cell lines clustered with the CDX tumours. The individual tumour replicates clustered closely together in all dimensions, with most tumours clustering separately from the cell lines in principal component 1 (Fig. S3). In general, the PDX models and UT‐SCC tumours clustered together by principal component 1, and were separate from the non‐HNSCC SiHa and HCT116/54C tumours and cells, as well as the widely studied FaDu line, which had the lowest CCN score of the HNSCC models.

### 3.2 Hypoxia in HNSCC tumour models by gene signatures

We next sought to characterise tumour hypoxia in our tumour models. We used RNAseq data from our tumour models and the TCGA database to evaluate expression of nine different hypoxia gene signatures that were generated specifically using HNSCC cell lines or tumours [31, 32, 33, 34, 35, 36] or from clinical tumours [37, 54, 55] (Fig. S4). The gene signatures all had varying expression of genes across the tumour samples, with replicate tumours typically clustering together. For many genes, the pattern of expression across the HNSCC tumour models did not reflect the variation observed across the TCGA primary tumour samples.

**Fig. 2:**
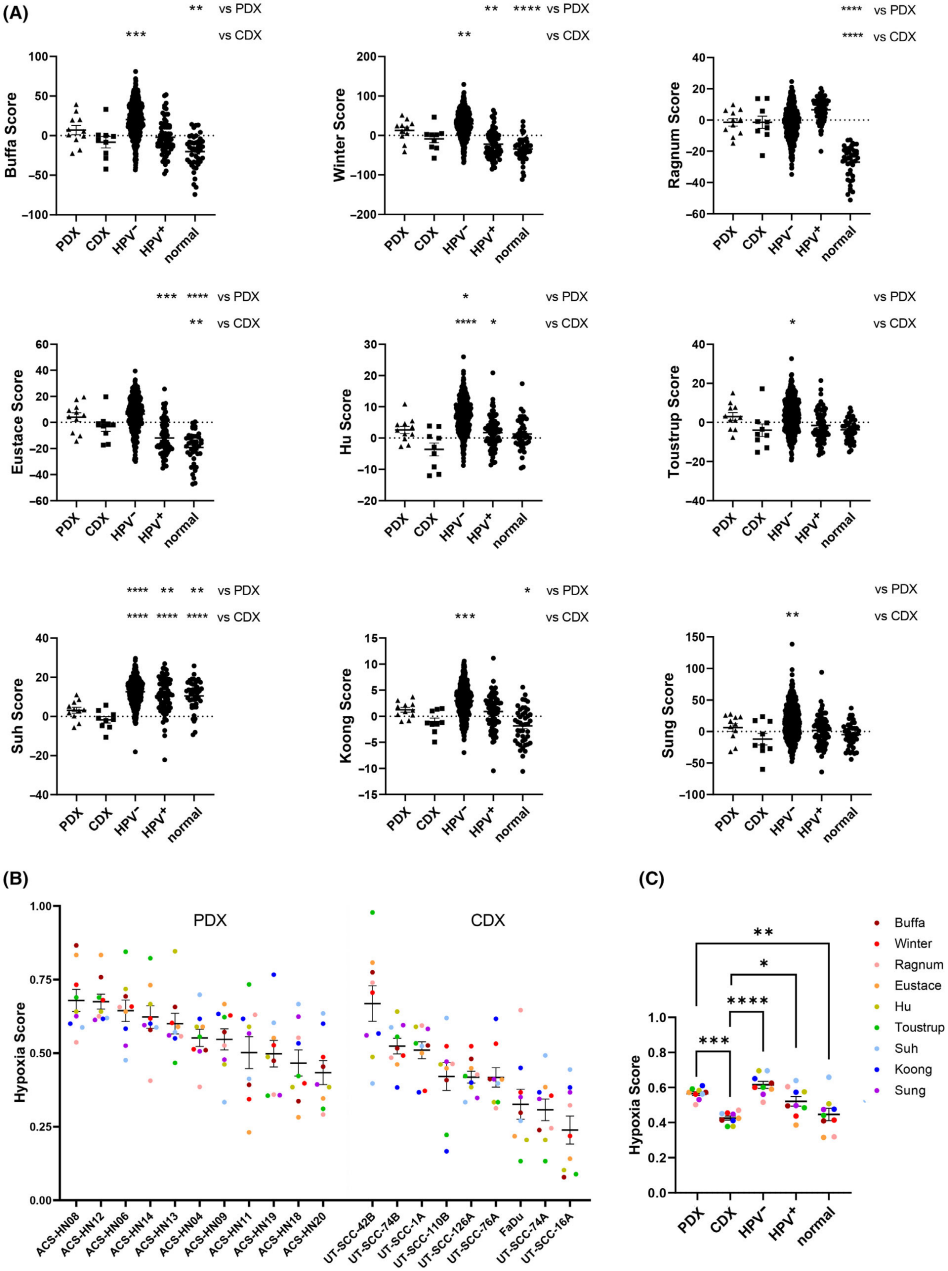
Evaluation of hypoxia in head and neck squamous cell carcinoma (HNSCC) tumour models using hypoxia gene signatures. (A) Signature Score for the nine hypoxia gene signatures in patient‐derived xenograft (PDX) tumours (*n* = 11), cell line‐derived xenograft (CDX) tumours (*n* = 9) and human papilloma virus (HPV)‐negative HNSCC (*n* = 447), HPV‐positive HNSCC (*n* = 73) and normal tissue samples (*n* = 44) from The Cancer Genome Atlas (TCGA). (B) Hypoxia score in individual HNSCC tumour models for the nine hypoxia gene signatures. Each datapoint represents the average hypoxia signature score of three tumours. (C) Hypoxia score in average HNSCC tumour models for the nine hypoxia gene signatures in comparison to the TCGA HPV‐negative HNSCC, HPV‐positive HNSCC and normal tissue samples. Each datapoint represents the average hypoxia signature score for the different sample types with *n* values as indicated in (A). Line and error bars represent mean ± SEM. *P < 0.05; **P < 0.01; ***P < 0.001; ****P < 0.0001 by one‐way ANOVA with Sidak's multiple comparison analysis of CDX and PDX to all other samples.

To allow for a comparison across HNSCC tumour models, we generated a signature score for each tumour sample based on whether the expression of each gene was above or below the median expression value across our xenograft samples. Replicate tumour samples had similar signature scores for all CDX and PDX tumour models (Fig. S5). Additionally, we evaluated RNAseq data from our cell lines grown under ambient oxygen and compared the hypoxia signature scores in these normoxic 2D cultures to their matched CDX models to determine the extent of gene expression reported by these hypoxia signatures under minimal hypoxia. For five signatures, CDX models had significantly higher scores than their paired cell lines (*P* < 0.05 for Hu and Suh signatures, *P* < 0.0005 for Eustace, Toustrup and Sung signatures), while for the remainder, the signature scores were only slightly higher (Buffa, Winter) or even slightly lower (Ragnum, Koong; Fig. S6). Next, we determined signature scores for primary HNSCC tumour and normal samples using RNAseq data from the TCGA dataset to allow a comparison of our 20 HNSCC tumour models to clinical samples (Fig. 2A). SiHa and HCT116/54C were excluded from these analyses, as they are not HNSCC. Overall, PDX tumours had higher signature scores than CDX tumours in all signatures except Ragnum. The highest expression on average was observed in the HPV‐negative primary HNSCC TCGA samples, while HPV‐positive samples had lower scores that were more similar to the xenograft samples, but higher than normal tissue samples.

The signature score from each gene signature was normalised to the number of genes in the signature to generate a Hypoxia Score and allow a direct comparison across all nine signatures for all 20 HNSCC tumour models (Fig. 2B, Fig. S7). The average Hypoxia Score across all nine signatures was significantly higher for PDX tumours than HNSCC CDX tumours (*P* < 0.001), and was similar to primary HNSCC TCGA samples, but significantly higher than TCGA normal samples (*P* < 0.01; Fig. 2C).

### 3.3 Hypoxia in HNSCC tumour models by pimonidazole

**Fig. 3:**
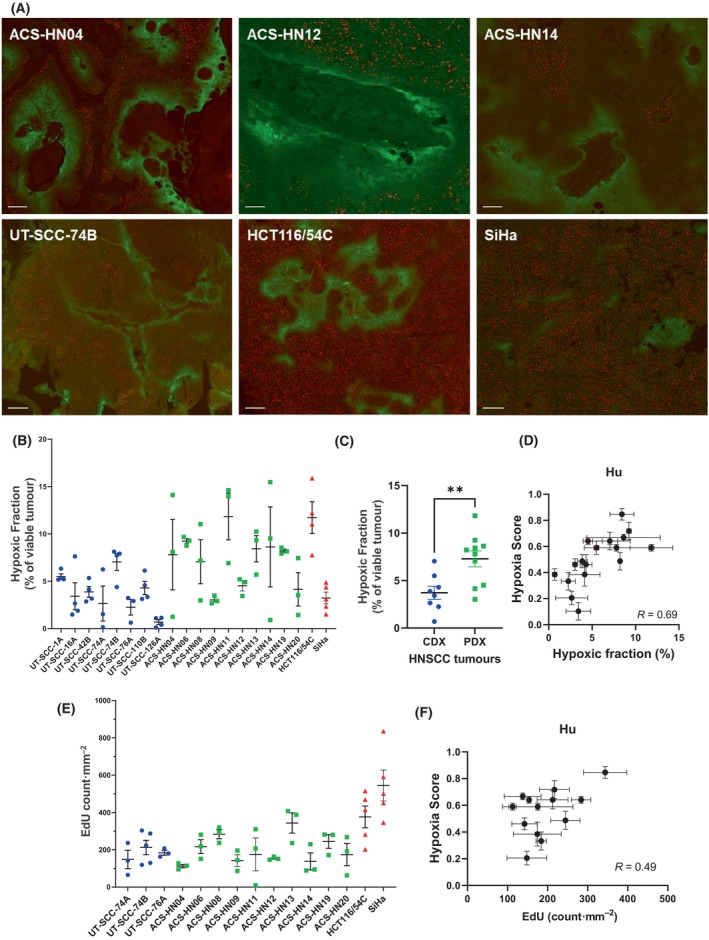
Evaluation of hypoxia and proliferation in patient‐derived xenograft (PDX) and cell line‐derived xenograft (CDX) tumour models using pimonidazole and 5‐ethynyl‐2′‐deoxyuridine (EdU) staining. (A) Representative images of pimonidazole and EdU staining in three PDX and three CDX tumour models. Green, pimonidazole; red, EdU. Scale bar = 200 μm. (B) Hypoxic fraction of individual tumour models (*n* = 3–5) and (C) average CDX (*n* = 8) and PDX (*n* = 10) head and neck squamous cell carcinoma (HNSCC) tumours as determined by the percentage of the viable region of the tumour that stained positive for pimonidazole. (D) Comparison of Hypoxia Score with hypoxic fraction for the Hu hypoxia gene signature across the CDX and PDX HNSCC tumours. (E) EdU count per mm^2^ of viable tumour area for individual CDX and PDX tumour models (*n* = 3–5). (F) Comparison of EdU count to Hypoxia Score for the Hu hypoxia gene signature across all HNSCC tumour models. Symbols in (B) and (E) represent a whole tumour section from an individual animal. Symbols in (C) represent average of 3–4 individual tumours for each tumour model. Line and error bars in (B), (C) and (E) represent mean ± SEM. **P < 0.01 by unpaired *T*‐test. Symbols and error bars represent mean ± SEM in (D) and (F) of 3–5 individual tumours. *R* values determined by Pearson correlation.

We have previously evaluated tumour hypoxia in large tumours (1000–1500 mm^3^) for some of our PDX models by pimonidazole immunohistochemistry [40]. Here, we extended this characterisation to our remaining PDX models as well as our CDX models, but evaluated hypoxia in small tumours at approximately the size when they are recruited for treatment (~ 250 mm^3^), rather than in large tumours. In certain models, we also evaluated tumour proliferation status by EdU staining. All tumours showed evidence of tumour hypoxia and proliferation by pimonidazole and EdU staining, respectively, that occurred in mutually exclusive regions of the tumour (Fig. 3A). Pimonidazole staining differed across tumour models (range from 0.69 ± 0.25% to 11.8 ± 2.5%) and was highly variable between individual tumours for both CDX and PDX models, with CV values above 50% for 8 of 19 tumour models (Fig. 3B). This is in contrast to the hypoxia gene signatures, where Hypoxia Score CV values did not exceed 50% in all 22 tumour models across six of the signatures and only exceeded 50% for 1–3 tumour models for the other three signatures. Despite the variability in pimonidazole staining, overall, the HNSCC PDX tumour models had a significantly higher average pimonidazole‐positive fraction of the tumour than the HNSCC CDX models did (*P* < 0.01; Fig. 3C), which combined with the higher hypoxia score of the PDX models (Fig. 2C), suggests that our HNSCC PDX tumour models were more hypoxic than our HNSCC CDX models. There were no strong correlations between hypoxic fraction by pimonidazole staining and hypoxia gene signatures across the HNSCC tumours (Fig. S8), with the Hu signature performing best (*R* = 0.69; Fig. 3D).

EdU staining was also quantitated and correlated to the hypoxia gene signatures, since several of the hypoxia gene signatures contain multiple proliferation‐related genes [31, 36, 54, 55]. EdU staining was less variable than hypoxia staining (CV > 50% in only 4 of 14 tumour models evaluated; Fig. 3E), but correlated poorly with most of the hypoxia gene signatures (Fig. S9), with the highest correlation again observed with the Hu signature (Fig. 3F).

### 3.4 Activity of HAPs in PDX and CDX tumour models

**Fig. 4:**
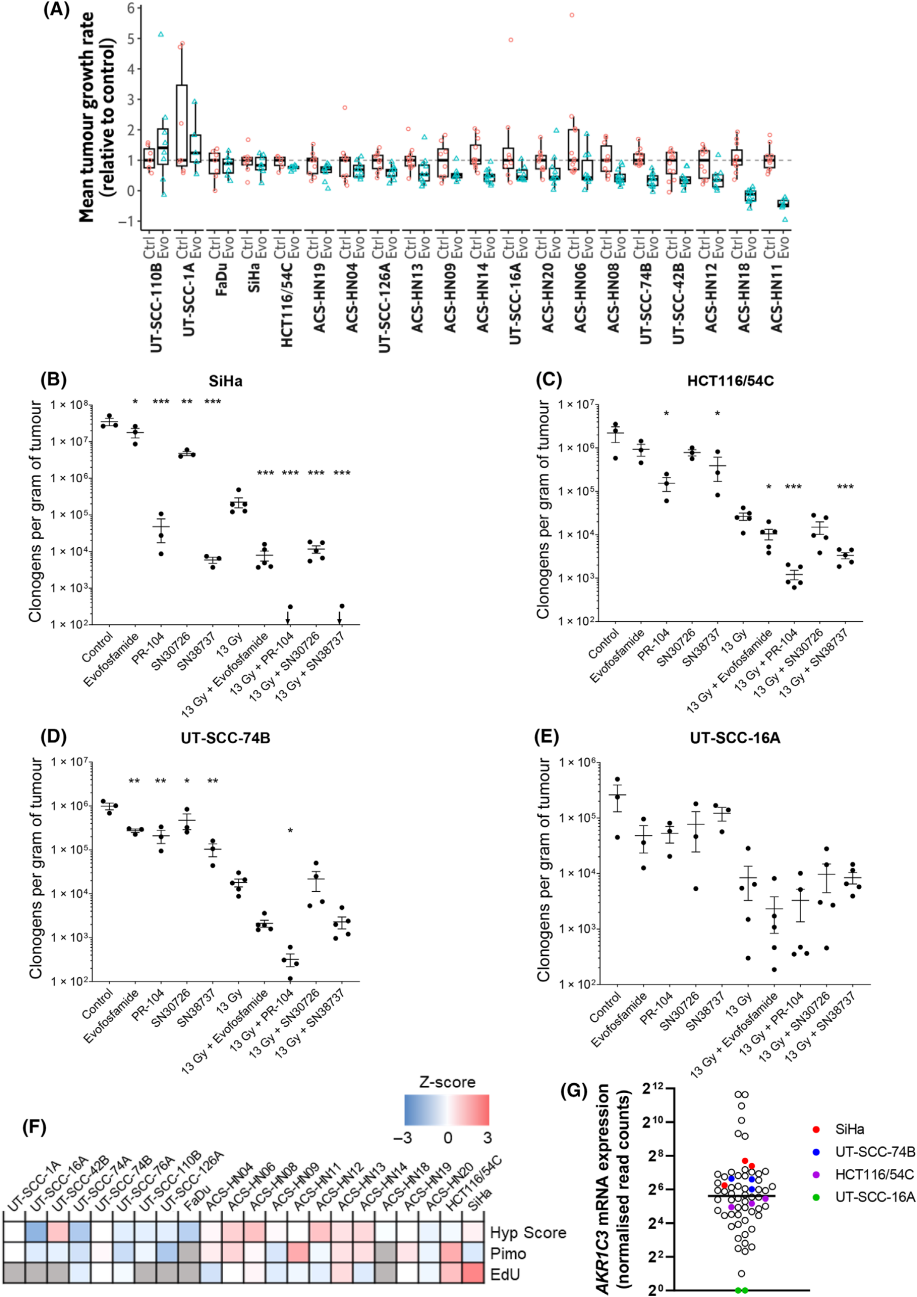
Targeting tumour models with hypoxia‐activated prodrugs (HAPs). (A) Relative mean daily growth rate of cell line‐derived xenograft (CDX) and patient‐derived xenograft (PDX) tumours treated with 50 mg·kg^−1^ evofosfamide (Evo) or saline (Ctrl) at qd × 5 for 3 weeks by IP injection (*n* = 5–12). Mean daily growth rates were normalised to the median value across control samples. Number of surviving clonogens per gram of tumour in *ex vivo* clonogenic assays in (B) SiHa, (C) HCT116/54C, (D) UT‐SCC‐74B and (E) UT‐SCC‐16A tumours from mice treated with 150 mg·kg^−1^ evofosfamide, 578 mg·kg^−1^ PR‐104, 40 mg·kg^−1^ SN30726 or SN38737 and/or whole‐body irradiation at 13 Gy (*n* = 3–5). *P < 0.05; **P < 0.01; ***P < 0.001 vs control for drug only or vs radiation for drug plus radiation by one‐way ANOVA with Dunnett's multiple comparison analysis. Arrows indicate 4 out of 5 colonies were below limit of detection. Line and error bars represent mean ± SEM. (F) Heatmap of *z*‐scores of different hypoxia and proliferation metrics determined by gene signatures or histochemistry across the 22 tumour models. Hyp Score = Hypoxia Score, Pimo = pimonidazole‐positive fraction. Grey = not determined. (G) AKR1C3 expression in CDX and PDX tumour models. Each symbol represents an individual tumour. Line represents the median.

In our earlier investigation, a significant but weak positive correlation was observed between pimonidazole staining and the antitumour efficacy of the hypoxia‐activated prodrug, evofosfamide, in 10 PDX models [40]. To determine if this correlation was maintained in a larger set of HNSCC tumour models, we evaluated the antitumour efficacy of evofosfamide across our three new PDX models and seven HNSCC CDX tumour models, with SiHa and HCT116/54C included for comparison (Fig. S10). The tumour take rate for UT‐SCC‐74A and ‐76A was too low for inclusion. Tumour growth data for 20 of these models were smoothed to reduce measurement variability by fitting Gompertz curves and the daily growth rates were estimated from these curves for the three‐week treatment period (ACS‐HN07 and ACS‐HN10 from our previous study [40] were excluded due to poor reproducibility of growth at P3). This allowed a direct comparison of individual evofosfamide‐treated and vehicle‐treated tumours (Fig. 4A). Significant differences in daily growth rate between control and treated tumours were observed for 5 of 20 tumour models (ACS‐HN08, ACS‐HN11, ACS‐HN14, ACS‐HN18 and UT‐SCC‐74B; *P* < 0.005, unpaired *t*‐test with Holm‐Sidak multiple comparison analysis). Next, we correlated mean daily growth rate following evofosfamide treatment with each hypoxia gene signature, as well as with pimonidazole and EdU staining to determine if hypoxia or proliferation markers could predict which tumour models were most sensitive to evofosfamide, but no significant relationships were observed (Fig. S11).

Since the assessment of antitumour efficacy in tumour xenograft models is not suited to evaluating the hypoxia dependence of HAPs, we next sought to evaluate the activity of evofosfamide and three other HAPs using an *ex vivo* tumour clonogenic assay that assesses the drug‐induced killing of hypoxic cells by utilising a high dose of radiation to eliminate oxic cells, such that surviving clonogens arise from the radioresistant hypoxic compartment. UT‐SCC‐16A, UT‐SCC‐74B. HCT116/54C and SiHa tumour models were selected as these were the only ones that were found to form stable single cell colonies with sufficient *ex vivo* plating efficiency, and growth as xenografts, to be suitable for this assay (Table S5). Mice were treated with one of four different HAPs and/or 13 Gy whole‐body irradiation, with the HAPs administered immediately after irradiation so that killing of hypoxic cells was induced by cytotoxicity of the released active drugs rather than radiosensitisation. Tumours were excised for clonogenic assay 18 h after treatment. The HAPs included evofosfamide and PR‐104, which both release DNA‐crosslinking nitrogen mustards [14, 56] and were treated at 75% of their maximum tolerated dose, as well as two nitroCBI DNA minor groove alkylating agent prodrugs: SN30726 and SN38737, that were tested at well‐tolerated doses equivalent to those found to inhibit tumour growth previously [20]. All four HAPs significantly reduced clonogenic survival of hypoxic cells in SiHa tumours, as indicated by fewer surviving clonogens when combined with 13 Gy radiation versus radiation alone (Fig. 4B). For PR‐104 and SN38737, surviving clonogens were below the level of detection in four out of five tumours. In HCT116/54C, PR‐104, SN38737 and evofosfamide significantly reduced clonogenic survival when combined with radiation versus radiation alone (Fig. 4C), while in UT‐SCC‐74B tumours only PR‐104 in combination with radiation significantly reduced clonogenic survival versus radiation alone, with evofosfamide and SN38737 causing large reductions of borderline statistical significance (*P* = 0.06; Fig. 4D). All four therapies also demonstrated significant single‐agent activity in SiHa and UT‐SCC‐74B tumours compared to controls, as did PR‐104 and SN38737 in HCT116/54C tumours (Fig. 4B–D). For evofosfamide, this single‐agent activity was consistent with its single‐agent antitumour efficacy in UT‐SCC‐74B tumours, but not for SiHa (Fig. 4A, Fig. S10). UT‐SCC‐16A tumours had the lowest *ex vivo* plating efficiency of the four tumour models, which likely contributed to the variable data and lack of significant differences in any treatment group (Fig. 4E).

On comparison across the 22 tumour models, SiHa had only a moderate average hypoxia score and pimonidazole‐positive hypoxic fraction (Fig. 4F), suggesting that hypoxia status alone could not explain the greater activity of the HAPs in this model. However, SiHa did have the highest EdU count. HCT116/54C had a high EdU count and pimonidazole‐positive hypoxic fraction, yet this model was less sensitive to hypoxia‐targeting therapies than SiHa and showed similar sensitivity to UT‐SCC‐74B, which had moderate hypoxia and proliferation (Fig. 4C,D). One possibility to explain the differences in the models is variability in expression of the reductase enzymes responsible for activating the HAPs. The importance of differences in expression of reductases is evident particularly for PR‐104, where mRNA for the two‐electron reductase AKR1C3, which catalyses its metabolic activation independently of hypoxia [21], was more highly expressed in SiHa > UT‐SCC‐74B > HCT116/54C tumours, with very little to no expression in UT‐SCC‐16A (Fig. 4F). UT‐SCC‐16A tumours had a low Hypoxia Score and minimal pimonidazole‐positive hypoxic fraction and had no clear treatment effect in the clonogenic assay (Fig. 4D).

## 4 Discussion

Tumour hypoxia negatively influences HNSCC patient prognosis and response to therapy, but also provides an opportunity for tumour‐selective therapeutic targeting. In this study, we characterised 20 HNSCC CDX and PDX tumour models (and two non‐HNSCC CDX models) for their fidelity to represent clinical HNSCC in gene expression, hypoxia status, proliferation, and sensitivity to hypoxia‐targeting agents.

Analysis of whole transcriptome gene expression data through the CancerCellNet computational tool revealed that our PDX tumours more closely resembled clinical HNSCC than CDX tumours or cell lines did. There are already various reports to show that PDX tumours more faithfully represent the gene expression of clinical tumours than CDX tumours and cell lines [7], despite typically lacking human stromal cells at early passages [57, 58, 59], but nonetheless, this confirms that our new PDX models provide clinically relevant models of HNSCC. Additionally, our HNSCC CDX tumour models had greater transcriptional fidelity for HNSCC than their paired cell lines as evaluated by CancerCellNet analysis. These findings indicate the transcriptional changes that occur as 2D cell lines grow as 3D tumours increase their similarity to clinical tumours and enhance the clinical relevance of xenograft models over 2D cell lines. A broad evaluation of the differentially expressed genes between paired cell lines and xenografts showed many genes being upregulated in CDX xenografts compared to 2D cell lines, with a smaller number being downregulated. Most of the same genes were also found to have changes in gene expression in the same direction in PDX tumours compared to cells. These patterns of gene expression changes suggest that growth as tumours, as opposed to 2D cultures, resulted in numerous genes being switched on. Although fewer genes were downregulated in tumours compared to cells, gene set tests revealed that these downregulated genes were enriched for those involved in certain metabolic processes including oxidative phosphorylation, DNA repair, and MYC targets, and may reflect cellular adaptations to the laboratory culturing conditions where oxygen and nutrients are plentiful and facilitate rapid cell growth.

Tumour hypoxia in our HNSCC models was evaluated both by hypoxia gene signatures and pimonidazole immunohistochemistry. On average, PDX tumours were more hypoxic than CDX tumours by both measures, despite high variability, particularly with pimonidazole staining, and a lack of strong correlations between the hypoxia gene signatures and pimonidazole or EdU staining across the tumour models. The extent of hypoxia in PDX models is not well characterised and so further investigation is required to confirm if this result is generalisable across other PDX/CDX models. Therefore, it remains unclear if the increased hypoxia in PDX versus CDX tumours observed in this study was inherent to PDX models, due to, for example, their greater heterogeneity [60] influencing vascularisation and/or oxygen consumption, or if it simply arose from differences in tumour formation and growth between subcutaneously injected cells (CDX models) and three passages of tumour fragments (PDX models). Additionally, there is conflicting evidence as to whether the hypoxia status of PDX tumours is consistent with patient tumours [40, 61, 62, 63] or is greater [64, 65]. Our data showing that the expression of hypoxia gene signatures in PDX tumours is largely consistent with HNSCC TCGA tumours provides evidence that subcutaneous PDX models can model clinical tumour hypoxia. Furthermore, the average pimonidazole‐positive hypoxic fractions observed for both PDX and CDX tumours in this study are within the range of those reported in HNSCC patients [66, 67, 68]. It should be noted, however, that oxygen consumption rates by the tumour parenchyma and the capacity of cancer cells to survive in decreased oxygenation will also impact tumour hypoxia, but these were not evaluated here.

The high variability in hypoxic fraction between individual tumours of the same model is consistent with our previous findings in large tumours [40] and in other tumour models [18, 69, 70, 71]. This variability, together with the small range of hypoxic fractions we have observed between different tumour models, here and previously [40], suggests that pimonidazole immunohistochemistry on its own is not sufficient for predicting sensitivity of xenografts to hypoxia‐targeting therapies. We also evaluated hypoxia gene signatures for this purpose, finding much less variability in Hypoxia Scores for individual signatures across different tumours of the same model, but still considerable variability between different signatures. Each of the signatures have been validated to predict hypoxia in either HNSCC models or clinical samples, so it was surprising to see large differences between the signatures. Additionally, although cognisant that the signatures have not been validated to report on hypoxia in normoxic cultures, we noted that four of the hypoxia signatures (Ragnum, Koong, Buffa and Winter) had moderate hypoxia signature scores in cell lines relative to their paired CDX tumours, despite the HNSCC cell lines being grown as monolayers in aerobic conditions and therefore being well oxygenated. This indicates that these hypoxia signatures include genes that can be expressed in the absence of hypoxia, e.g. proliferation genes [55] and highlights the difficulty in detecting hypoxia using a gene expression signature, which will inevitably pick up pseudohypoxia or gene expression changes that mimic hypoxia [10], e.g. those related to metabolic reprogramming [72, 73, 74], while also likely being dominated by mildly hypoxic signals rather than the more severe hypoxia detected by pimonidazole [10]. Furthermore, any contribution of stromal (particularly endothelial and immune) cells to expression of hypoxia‐related genes in clinical tumours is lost in the PDX and CDX tumours as mouse reads were excluded during the sequencing pipeline. With so many hypoxia gene signatures now reported in addition to those described in this study [75], the question also remains which signature is the most appropriate to use to predict sensitivity. As various hypoxia‐responsive genes are induced at different oxygenation states [76], each signature may be providing a slightly different measure by assessing overlapping, but slightly distinct, subsets of cells. The Hu signature showed the strongest correlations to pimonidazole or EdU staining, while the Toustrup, Eustace and Sung signatures diverged the most between CDX tumours and their paired cell lines. Rather than attempting to select an optimal signature, our approach was to use the average score across multiple signatures to minimise the variability, but this may still be compromised by poorly performing signatures.

Since two separate methods were used to evaluate tumour hypoxia, we tested the relationship between hypoxia gene signature scores and pimonidazole‐positive hypoxic fraction across our tumour models, with only moderate to weak correlations observed for the nine signatures, as we have seen previously for the Toustrup signature [40]. The lack of strong correlation between two different methods to assess hypoxia was not unexpected [77, 78, 79] and is likely due to numerous factors. Such factors include the mild degree of hypoxia required to activate HIF‐regulated gene expression compared to severe hypoxia needed for pimonidazole staining [10], the requirement for pimonidazole activation by suitable one‐electron reductases, which likely vary in expression between tumours, the low range in average hypoxic fractions across the tumour models and any impact of necrotic cells, which were excluded from hypoxic fraction determinations, on gene expression. Therefore, despite the lack of correlation, both methods are still appropriate biomarkers of hypoxia, although the choice of hypoxia marker should reflect the application, depending, in particular, on the mechanism of action of any hypoxia‐targeting (or hypoxia‐sparing) agent [10]. Since proliferation is also inherent to many hypoxia signatures and can influence response to hypoxia‐targeting therapies [13, 55, 80], we also evaluated proliferation by EdU staining, which again did not strongly correlate with hypoxia gene signatures.

Various hypoxia‐targeting strategies have advanced to the clinic but failed in part because of a lack of predictive biomarkers to preselect suitable patients [15]. Therefore, we sought to determine if hypoxia gene signatures, pimonidazole staining or EdU staining in our tumour models could predict sensitivity to HAPs by either tumour growth inhibition or the killing of hypoxic cells in an *ex vivo* clonogenic assay. Overall, there was no obvious pattern to suggest that hypoxia or proliferation markers alone could determine therapeutic activity of HAPs. This is consistent with previous studies, which at best report weak correlations between HAP efficacy and hypoxia status in preclinical models [17, 40, 81] and suggests that hypoxia (or proliferation) alone is not sufficient to predict sensitivity. Other factors, including the expression of activating reductase enzymes (e.g. AKR1C3 for PR‐104) and intrinsic sensitivity to the active metabolites (e.g. DNA damage repair status for DNA‐reactive HAPs) [82], likely also play important roles and can improve predictions of HAP sensitivity when combined with hypoxia biomarkers [17, 18]. The recent development of a DNA‐methylome–based tumour hypoxia classifier [83] provides an alternative approach for assessing hypoxia that may supplement the methods used here, although it is yet to be investigated in preclinical models and may be sensitive to pseudohypoxia.

## 5 Conclusions

In summary, we generated new preclinical models of HNSCC with greater transcriptional fidelity for clinical HNSCC tumours than HNSCC cell lines. We evaluated the hypoxia and proliferation status of the tumour models, showing that the PDX tumours were on average more hypoxic than CDX tumours. Hypoxia or proliferation status alone could not determine sensitivity of models for targeting of hypoxic cells by HAPs by either tumour growth inhibition or killing of hypoxic cells in an *ex vivo* clonogenic assay. Optimal biomarkers for HAPs will need to incorporate multiple determinants of sensitivity, of which hypoxia is just one aspect and is complemented by reductase expression and biomarkers for intrinsic sensitivity to the active metabolites.

## Conflict of interest

The authors declare no conflict of interest.

## Author contributions

Conceptualisation: TWL, DCS, MT, FBP, AMJM, FWH, WRW and SMFJ; data curation: TWL and SMFJ; funding acquisition: WRW and SMFJ; Investigation and methodology: TWL, JKH, ML, SPM, AL and SMFJ; Resources: TWL, MT, FBP, AMJM, FWH and SMFJ; Visualisation: TWL, DCS and SMFJ; Supervision: SMFJ; Writing – original draft: TWL, DCS and SMFJ; Writing – review and editing: all authors.

## Supporting information


**Fig. S1.** CancerCellNet (CCN) Scores for tumour types other than HNSCC that were common in the HNSCC tumour models and cell lines.
**Fig. S2.** Heatmap of CCN HNSCC diagnostic genes across cells, CDX and PDX tumours in comparison to TCGA Primary and TCGA Normal samples.
**Fig. S3.** Principal component analysis for the log_2_ normalised counts per million of the 500 most variable genes for PDX and CDX tumours and cell lines.
**Fig. S4.** Heatmaps of gene expression for the genes used in the nine hypoxia gene signatures.
**Fig. S5.** Signature scores for the nine hypoxia gene signatures for individual CDX and PDX tumours.
**Fig. S6.** Comparison of Signature scores for the nine hypoxia gene signatures for paired CDX tumours and cell lines.
**Fig. S7.** Hypoxia Scores for the non‐HNSCC CDX tumours.
**Fig. S8.** Comparison of Hypoxia Score and hypoxic fraction for HNSCC tumours for the nine hypoxia gene signatures.
**Fig. S9.** Comparison of Hypoxia Score and EdU count for HNSCC tumours for the nine hypoxia gene signatures.
**Fig. S10.** Tumour growth curves in mice with PDX or CDX tumours treated with 50 mg/kg evofosfamide in saline or control vehicle by IP injection at qd × 5 for 3 weeks.
**Fig. S11.** Comparison of evofosfamide daily growth rate with a) Hypoxia Score, b) hypoxic fraction and c) EdU count for HNSCC tumour models.
**Table S1.** STR profiles of UT‐SCC‐54A, UT‐SCC‐54B, UT‐SCC‐54C and HCT116.
**Table S2.** Gene mutations in HCT116/54C cells.
**Table S3.** RNASeq count matrix for cell, CDX and PDX samples.
**Table S4.** VirTect viral RNA results for HPV status.
**Table S5.** Clonogenic assay plating efficiencies.

## Data Availability

RNASeq count data generated during this study are included in Table S3. Other RNASeq data analysed in this study are publicly available from the repositories listed in the methods. Source code and datasets derived from the analyses of these original datasets are available from the corresponding author on reasonable request.
