# Supplementary material for: Clinical relevance and therapeutic predictive ability of hypoxia biomarkers in head and neck cancer tumour models
Source: Mol Oncol. 2024 Mar 1;18(8):1885–903. doi: 10.1002/1878-0261.13620 (PMC11306523; doi:10.1002/1878-0261.13620)
Supplement: Supplementary file 1 — Fig. S1. CancerCellNet (CCN) Scores for tumour types other than HNSCC that were common in the HNSCC tumour models and cell lines. Fig. S2. Heatmap of CCN HNSCC diagnostic genes across cells, CDX and PDX tumours in comparison to TCGA Primary and TCGA Normal samples. Fig. S3. Principal component analysis for the log2 normalised counts per million of the 500 most variable genes for PDX and CDX tumours and cell lines. Fig. S4. Heatmaps of gene expression for the genes used in the nine hypoxia gene signatures. Fig. S5. Signature scores for the nine hypoxia gene signatures for individual CDX and PDX tumours. Fig. S6. Comparison of Signature scores for the nine hypoxia gene signatures for paired CDX tumours and cell lines. Fig. S7. Hypoxia Scores for the non‐HNSCC CDX tumours. Fig. S8. Comparison of Hypoxia Score and hypoxic fraction for HNSCC tumours for the nine hypoxia gene signatures. Fig. S9. Comparison of Hypoxia Score and EdU count for HNSCC tumours for the nine hypoxia gene signatures. Fig. S10. Tumour growth curves in mice with PDX or CDX tumours treated with 50 mg/kg evofosfamide in saline or control vehicle by IP injection at qd × 5 for 3 weeks. Fig. S11. Comparison of evofosfamide daily growth rate with a) Hypoxia Score, b) hypoxic fraction and c) EdU count for HNSCC tumour models. Table S1. STR profiles of UT‐SCC‐54A, UT‐SCC‐54B, UT‐SCC‐54C and HCT116. Table S2. Gene mutations in HCT116/54C cells. Table S3. RNASeq count matrix for cell, CDX and PDX samples. Table S4. VirTect viral RNA results for HPV status. Table S5. Clonogenic assay plating efficiencies. [file MOL2-18-1885-s001.zip › mol213620-sup-0001-Supinfo.docx]

**Supplementary Tables and Figures for:**

**Clinical relevance and therapeutic predictive ability of hypoxia biomarkers in head and neck cancer tumour models**

Tet Woo Lee^1,2^, Dean C. Singleton^1,2,3^, Julia K. Harms^1^, Man Lu^1^, Sarah P. McManaway^1^, Amy Lai^1,4^, Moana Tercel^1,2^, Frederik B. Pruijn^1,2^, Andrew M.J. Macann^5^, Francis W. Hunter^1,2,6^, William R. Wilson^1,2^, Stephen M.F. Jamieson^1,2,4*^

¹Auckland Cancer Society Research Centre, University of Auckland, Auckland, New Zealand.

²Maurice Wilkins Centre for Molecular Biodiscovery, University of Auckland, Auckland, New Zealand.

^3^Department of Molecular Medicine and Pathology, University of Auckland, Auckland, New Zealand.

^4^Department of Pharmacology and Clinical Pharmacology, University of Auckland, Auckland, New Zealand.

^5^Department of Radiation Oncology, Auckland City Hospital, Auckland, New Zealand.

^6^Oncology Therapeutic Area, Janssen Research and Development, Spring House, PA, USA

*Correspondence to: Stephen Jamieson ([s.jamieson@auckland.ac.nz](mailto:s.jamieson@auckland.ac.nz))

**Supplementary Results**

HCT116/54C cells (referred to as UT-SCC-54C in our earlier study [1]) were characterised to determine if they were a mixed culture of HCT116 and UT-SCC-54C cells or have been outgrown by either line. The STR profile of HCT116/54C was compared with the profiles of HCT116 and original UT-SCC-54C cultures as well as UT-SCC-54A and UT-SCC-54B, which came from the same patient as UT-SCC-54C [1]. HCT116/54C cells had a separate STR profile to the original UT-SCC-54C culture as well as to UT-SCC-54A and UT-SCC-54B, which were identical, and instead showed up to 88% similarity to the STR profile of HCT116 using the CLASTR 1.4.4 STR similarity profiler (Table S1). Re-analysis of our published exome sequencing data for HCT116/54C cells [1] on COSMIC v96 [2] indicated that of the 147 mutations that were identified in UT-SCC-54C cells, 120 were shared with HCT116 cells (COSS1998442 and COSS2301978), while only four were detected in UT-SCC-54A and UT-SCC-54B (Table S2). For comparison, 22 mutations were shared between UT-SCC-54A and UT-SCC-54B out of the 26 mutations detected in UT-SCC-54A and 34 in UT-SCC-54B [1]. Our earlier investigation also determined the ploidy status of our cell lines. HCT116/54C cells were like HCT116 cells [3] found to be near diploid, while UT-SCC-54A and UT-SCC-54B were mixed ploidy, but predominantly (>97%) hypotetraploid [1]. Combined these data strongly suggest that although our HCT116/54C cultures were initially a mixed culture of UT-SCC-54C and HCT116 cells, they have been outgrown by HCT116 and are now essentially HCT116 cells. Additionally, as UT-SCC-54A and -54B cells do not form tumours in mice [4], but HCT116 cells do [5], we expect all cells in our HCT116/54C tumour model to be HCT116 cells rather than UT-SCC-54C and therefore consider HCT116/54C tumours to be a model of HCT116 rather than a mixed culture tumour model. Further evidence to support this is that the histopathology and pimonidazole imaging of HCT116/54C tumours [1], closely resembles HCT116 tumours [6–8] and lacks the SCC morphology of other UT-SCC tumours [1,9,10].

**Supplementary Acknowledgement:** We thank Prof. Bradly Wouters for sharing STR data on the original stocks of early-passage UT-SCC-54C cultures.

**Supplementary References:**

1 Jamieson SMF, Tsai P, Kondratyev MK, Budhani P, Liu A, Senzer NN, Chiorean EG, Jalal SI, Nemunaitis JJ, Kee D, Shome A, Wong WW, Li D, Poonawala-Lohani N, Kakadia PM, Knowlton NS, Lynch CRH, Hong CR, Lee T-W, Grénman RA, Caporiccio L, McKee TD, Zaidi M, Butt S, Macann AMJ, McIvor NP, Chaplin JM, Hicks KO, Bohlander SK, Wouters BG, Hart CP, Print CG, Wilson WR, Curran MA & Hunter FW (2018). Evofosfamide for the treatment of human papillomavirus-negative head and neck squamous cell carcinoma. *JCI Insight*. **3**, e122204.

2 Tate JG, Bamford S, Jubb HC, Sondka Z, Beare DM, Bindal N, Boutselakis H, Cole CG, Creatore C, Dawson E, Fish P, Harsha B, Hathaway C, Jupe SC, Kok CY, Noble K, Ponting L, Ramshaw CC, Rye CE, Speedy HE, Stefancsik R, Thompson SL, Wang S, Ward S, Campbell PJ & Forbes SA (2019). COSMIC: the Catalogue Of Somatic Mutations In Cancer. *Nucleic Acids Res*. **47**, D941–D947.

3 Lengauer C, Kinzler KW & Vogelstein B (1997). Genetic instability in colorectal cancers. *Nature*. **386**, 623–627.

4 Lee TW, Hunter FW, Tsai P, Print CG, Wilson WR & Jamieson SMF (2023). Clonal dynamics limits detection of selection in tumour xenograft CRISPR/Cas9 screens. *Cancer Gene Ther*. 1–14.

5 Bonnet M, Hong CR, Wong WW, Liew LP, Shome A, Wang J, Gu Y, Stevenson RJ, Qi W, Anderson RF, Pruijn FB, Wilson WR, Jamieson SMF, Hicks KO & Hay MP (2018). Next-Generation Hypoxic Cell Radiosensitizers: Nitroimidazole Alkylsulfonamides. *J Med Chem*. **61**, 1241–1254.

6 Gali-Muhtasib H, Ocker M, Kuester D, Krueger S, El-Hajj Z, Diestel A, Evert M, El-Najjar N, Peters B, Jurjus A, Roessner A & Schneider-Stock R (2008). Thymoquinone reduces mouse colon tumor cell invasion and inhibits tumor growth in murine colon cancer models. *J Cell Mol Med*. **12**, 330–342.

7 Geagea AG, Rizzo M, Jurjus A, Cappello F, Leone A, Tomasello G, Gracia C, Kattar SA, Massaad-Massade L & Eid A (2019). A novel therapeutic approach to colorectal cancer in diabetes: role of metformin and rapamycin. *Oncotarget*. **10**, 1284–1305.

8 Endo H & Inoue M (2019). Dormancy in cancer. *Cancer Sci*. **110**, 474–480.

9 Viiklepp K, Nissinen L, Ojalill M, Riihilä P, Kallajoki M, Meri S, Heino J & Kähäri V-M (2022). C1r Upregulates Production of Matrix Metalloproteinase-13 and Promotes Invasion of Cutaneous Squamous Cell Carcinoma. *J Invest Dermatol*. **142**, 1478-1488.e9.

10 Riihilä P, Viiklepp K, Nissinen L, Farshchian M, Kallajoki M, Kivisaari A, Meri S, Peltonen J, Peltonen S & Kähäri V ‐M. (2020). Tumour‐cell‐derived complement components C1r and C1s promote growth of cutaneous squamous cell carcinoma. *Br J Dermatol*. **182**, 658–670.

11 Harms JK, Lee T-W, Wang T, Lai A, Kee D, Chaplin JM, McIvor NP, Hunter FW, Macann AMJ, Wilson WR & Jamieson SMF (2019). Impact of Tumour Hypoxia on Evofosfamide Sensitivity in Head and Neck Squamous Cell Carcinoma Patient-Derived Xenograft Models. *Cells*. **8**, 717.

**Table S1** STR profiles of UT-SCC-54A, UT-SCC-54B, UT-SCC-54C and HCT116

|  | HCT116/54C^1^ | UT-SCC-54C^2^ | UT-SCC-54A^1^ | UT-SCC-54B^1^ | HCT116^2^ |
| --- | --- | --- | --- | --- | --- |
| TH01 | 8,9 | 9.3 | 9.3 | 9.3 | 8,9 |
| D3S1358 | 12,19 | 17 | 17 | 17 | 12,18,19 |
| vWa | 16,22 | 14,18 | 14,18 | 14,18 | 17,22 |
| D21S11 | 29,30 | 31 | 31,32.2 | 31,32.2 | 29,30 |
| TPOX | 8 | 8,9 | 8,9 | 8,9 | 8 |
| D7S820 | 11,12 | 12 | 12 | 12 | 11,12 |
| D19S433 | 12,13 | 14 | 14 | 14 | 12,13 |
| D5S818 | 10,11 | 11 | 11 | 11 | 10,11 |
| D2S1338 | 16 | 20,24 | 20,24 | 20,24 | 16 |
| D16S539 | 11,13 | 11,12 | 11,12 | 11,12 | 11,13 |
| CSF1PO | 7,11 | 10 | 10 | 10 | 7,10 |
| D13S317 | 11,12 | 12 | 12 | 12 | 10,12 |
| FGA | 18,23 | 21,26 | 26 | 21,26 | 18,23 |
| D18S51 | 16,17 | 14,16 | 14,16 | 16 | 17 |
| D8S1179 | 12,14 | 10,14 | 10,14 | 10,14 | 12,14 |
| Amelogenin | X | X | X | X | X |

^1^ from Jamieson et al., 2018 [1]; HCT116/54C were referred to as UT-SCC-54C

^2^ from early-passage UT-SCC-54C cells profiled in Prof Bradly Wouters lab (University Health Network, Toronto, Canada)

^3^ from HCT116 cells (RRID: CVCL_0291) cultured in house in αMEM + 5% FCS

**Table S4** VirTect viral RNA results for HPV status. GI (GenInfo Identifier) and description of HPV viral genome with largest number of reads aligned to unmapped RNASeq reads shown

| **Model** | **Sample** | **GI** | **Description** | **Aligned reads** | **Continuous aligned length** | **Called HPV status** |
| --- | --- | --- | --- | --- | --- | --- |
| **1A** | cells | 1491683 | Human papillomavirus 72 (HPV72), complete genome | 76 | 77 | negative |
|  | T1 | 1491683 | Human papillomavirus 72 (HPV72), complete genome | 205 | 72 | negative |
|  | T2 | 1491683 | Human papillomavirus 72 (HPV72), complete genome | 201 | 83 | negative |
|  | T3 | 1491683 | Human papillomavirus 72 (HPV72), complete genome | 274 | 77 | negative |
| **16A** | cells | 1491683 | Human papillomavirus 72 (HPV72), complete genome | 116 | 85 | negative |
|  | T1 | 1491683 | Human papillomavirus 72 (HPV72), complete genome | 156 | 75 | negative |
|  | T2 | 1491683 | Human papillomavirus 72 (HPV72), complete genome | 142 | 76 | negative |
|  | T3 | 1491683 | Human papillomavirus 72 (HPV72), complete genome | 272 | 81 | negative |
| **42B** | cells | 1491683 | Human papillomavirus 72 (HPV72), complete genome | 82 | 74 | negative |
|  | T1 | 1491683 | Human papillomavirus 72 (HPV72), complete genome | 216 | 76 | negative |
|  | T2 | 1491683 | Human papillomavirus 72 (HPV72), complete genome | 323 | 85 | negative |
|  | T3 | 1491683 | Human papillomavirus 72 (HPV72), complete genome | 106 | 69 | negative |
| **74A** | cells | 1491683 | Human papillomavirus 72 (HPV72), complete genome | 76 | 83 | negative |
|  | T1 | 1491683 | Human papillomavirus 72 (HPV72), complete genome | 247 | 77 | negative |
|  | T2 | 1491683 | Human papillomavirus 72 (HPV72), complete genome | 430 | 74 | negative |
|  | T3 | 1491683 | Human papillomavirus 72 (HPV72), complete genome | 391 | 76 | negative |
| **74B** | cells | 1491683 | Human papillomavirus 72 (HPV72), complete genome | 68 | 72 | negative |
|  | T1 | 1491683 | Human papillomavirus 72 (HPV72), complete genome | 107 | 75 | negative |
|  | T2 | 1491683 | Human papillomavirus 72 (HPV72), complete genome | 84 | 85 | negative |
|  | T3 | 1491683 | Human papillomavirus 72 (HPV72), complete genome | 417 | 84 | negative |
| **76A** | cells | 1491683 | Human papillomavirus 72 (HPV72), complete genome | 94 | 85 | negative |
|  | T1 | 60955 | Human papillomavirus 6 (HPV6), complete genome | 555 | 97 | negative |
|  | T2 | 1491683 | Human papillomavirus 72 (HPV72), complete genome | 1247 | 84 | negative |
|  | T3 | 1491683 | Human papillomavirus 72 (HPV72), complete genome | 2038 | 85 | negative |
| **110B** | cells | 1491683 | Human papillomavirus 72 (HPV72), complete genome | 67 | 74 | negative |
|  | T1 | 1491683 | Human papillomavirus 72 (HPV72), complete genome | 70 | 71 | negative |
|  | T2 | 1491683 | Human papillomavirus 72 (HPV72), complete genome | 84 | 71 | negative |
|  | T3 | 1491683 | Human papillomavirus 72 (HPV72), complete genome | 304 | 80 | negative |
| **126A** | cells | 1491683 | Human papillomavirus 72 (HPV72), complete genome | 92 | 83 | negative |
|  | T1 | 1491683 | Human papillomavirus 72 (HPV72), complete genome | 608 | 85 | negative |
|  | T2 | 1491683 | Human papillomavirus 72 (HPV72), complete genome | 87 | 81 | negative |
|  | T3 | 1491683 | Human papillomavirus 72 (HPV72), complete genome | 259 | 83 | negative |
| **FaDu** | CCLE | 1491683 | Human papillomavirus 72 (HPV72), complete genome | 38 | 85 | negative |
|  | cells | 1491683 | Human papillomavirus 72 (HPV72), complete genome | 48 | 76 | negative |
|  | T1 | 1491683 | Human papillomavirus 72 (HPV72), complete genome | 467 | 75 | negative |
|  | T2 | 1491683 | Human papillomavirus 72 (HPV72), complete genome | 334 | 76 | negative |
|  | T3 | 1491683 | Human papillomavirus 72 (HPV72), complete genome | 633 | 81 | negative |
| **SiHa** | CCLE | 333031 | Human papillomavirus 16 (HPV16), complete genome | 7875 | 3098 | **positive** |
|  | T1 | 333031 | Human papillomavirus 16 (HPV16), complete genome | 7298 | 3133 | **positive** |
|  | T2 | 333031 | Human papillomavirus 16 (HPV16), complete genome | 11205 | 3065 | **positive** |
|  | T3 | 333031 | Human papillomavirus 16 (HPV16), complete genome | 10268 | 3051 | **positive** |
| **HCT116/ 54C** | cells | 1491683 | Human papillomavirus 72 (HPV72), complete genome | 98 | 73 | negative |
|  | T1 | 1491683 | Human papillomavirus 72 (HPV72), complete genome | 78 | 70 | negative |
|  | T2 | 1491683 | Human papillomavirus 72 (HPV72), complete genome | 48 | 71 | negative |
|  | T3 | 1491683 | Human papillomavirus 72 (HPV72), complete genome | 78 | 87 | negative |
| **HN04** | T1 | 1491683 | Human papillomavirus 72 (HPV72), complete genome | 278 | 68 | negative |
|  | T2 | 1491683 | Human papillomavirus 72 (HPV72), complete genome | 465 | 86 | negative |
|  | T3 | 1491683 | Human papillomavirus 72 (HPV72), complete genome | 440 | 86 | negative |
| **HN06** | T1 | 1491683 | Human papillomavirus 72 (HPV72), complete genome | 12 | 70 | negative |
|  | T2 | 1491683 | Human papillomavirus 72 (HPV72), complete genome | 439 | 77 | negative |
|  | T3 | 1491683 | Human papillomavirus 72 (HPV72), complete genome | 690 | 73 | negative |
| **HN08** | T1 | 1491683 | Human papillomavirus 72 (HPV72), complete genome | 755 | 78 | negative |
|  | T2 | 1491683 | Human papillomavirus 72 (HPV72), complete genome | 16 | 69 | negative |
|  | T3 | 1491683 | Human papillomavirus 72 (HPV72), complete genome | 373 | 71 | negative |
| **HN09** | T1 | 1491683 | Human papillomavirus 72 (HPV72), complete genome | 34 | 76 | negative |
|  | T2 | 1491683 | Human papillomavirus 72 (HPV72), complete genome | 22 | 70 | negative |
|  | T3 | 1491683 | Human papillomavirus 72 (HPV72), complete genome | 15 | 70 | negative |
| **HN11** | T1 | 397005 | Human papillomavirus 3 (HPV3), complete genome | 12 | 126 | negative |
|  | T2 | 1491683 | Human papillomavirus 72 (HPV72), complete genome | 12 | 74 | negative |
|  | T3 | 1491683 | Human papillomavirus 72 (HPV72), complete genome | 13 | 82 | negative |
| **HN12** | T1 | 1491683 | Human papillomavirus 72 (HPV72), complete genome | 33 | 72 | negative |
|  | T2 | 1491683 | Human papillomavirus 72 (HPV72), complete genome | 96 | 75 | negative |
|  | T3 | 1491683 | Human papillomavirus 72 (HPV72), complete genome | 39 | 72 | negative |
| **HN13** | T1 | 1491683 | Human papillomavirus 72 (HPV72), complete genome | 431 | 75 | negative |
|  | T2 | 1491683 | Human papillomavirus 72 (HPV72), complete genome | 478 | 73 | negative |
|  | T3 | 6970427 | Human papillomavirus 82 (HPV82), complete genome | 597 | 72 | negative |
| **HN14** | T1 | 9627389 | Human papillomavirus 7 (HPV7), complete genome | 28 | 74 | negative |
|  | T2 | 1491683 | Human papillomavirus 72 (HPV72), complete genome | 627 | 72 | negative |
|  | T3 | 1491683 | Human papillomavirus 72 (HPV72), complete genome | 734 | 73 | negative |
| **HN18** | T1 | 9627389 | Human papillomavirus 7 (HPV7), complete genome | 38 | 80 | negative |
|  | T2 | 1491683 | Human papillomavirus 72 (HPV72), complete genome | 156 | 74 | negative |
|  | T3 | 1491683 | Human papillomavirus 72 (HPV72), complete genome | 260 | 82 | negative |
| **HN19** | T1 | 1491683 | Human papillomavirus 72 (HPV72), complete genome | 497 | 77 | negative |
|  | T2 | 1491683 | Human papillomavirus 72 (HPV72), complete genome | 358 | 77 | negative |
|  | T3 | 1491683 | Human papillomavirus 72 (HPV72), complete genome | 549 | 73 | negative |
| **HN20** | T1 | 1491683 | Human papillomavirus 72 (HPV72), complete genome | 478 | 73 | negative |
|  | T2 | 1491683 | Human papillomavirus 72 (HPV72), complete genome | 7 | 68 | negative |
|  | T3 | 1491683 | Human papillomavirus 72 (HPV72), complete genome | 20 | 69 | negative |

**Table S5** Clonogenic assay plating efficiencies

| **Cell line** | **Plating Efficiency** | |
| --- | --- | --- |
|  | In vitro | Ex vivo |
| UT-SCC-1A | No colonies formed | - |
| UT-SCC-16A | 0.26 | 0.0066 |
| UT-SCC-42B | 0.09 | 0.018, tumours ulcerate |
| UT-SCC-74A | 0.35 | 0.026, low tumour take rate |
| UT-SCC-74B | 0.1 | 0.035 |
| UT-SCC-76A | No colonies formed | - |
| UT-SCC-110B | 0.07 | 0.00081 |
| UT-SCC-126A | 0.09 | 0.0036, sudden weight loss |
| FaDu | 0.25 | 0.020, low quality colonies |
| HCT116/54C | 0.53 | 0.069 |
| SiHa | 0.81 | 0.22 |

**Fig. S1** CancerCellNet (CCN) Scores for tumour types other than HNSCC that were common in the HNSCC tumour models and cell lines. Lines and error bars represent mean ± SEM. *, P<0.05; **, P<0.01; ****; P<0.0001; ns, nonsignificant by one-way ANOVA with Sidak's multiple comparison analysis


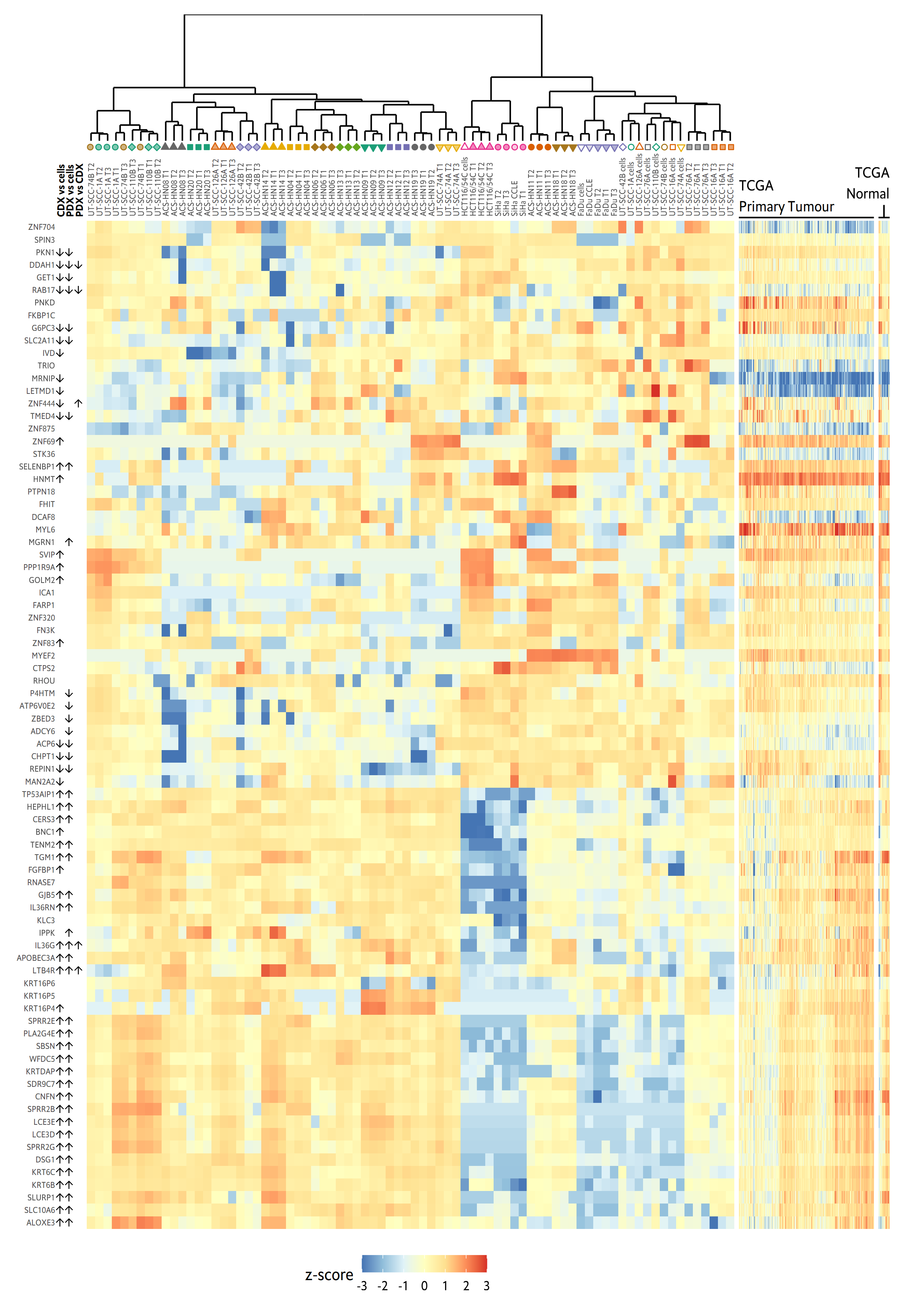


**Fig. S2** Heatmap of CCN HNSCC diagnostic genes (i.e. genes that contribute to the gene pairs used for the CCN HNSCC score) across cells, CDX and PDX tumours in comparison to TCGA Primary and TCGA Normal samples. Z-scores were calculated using mean and standard deviation of non-TCGA samples and limited to the range of -3 to +3. Samples within each subset and genes were hierarchically clustered by Euclidean distance using the Ward D2 method based on Z-score. ↑ (increased expression) and ↓ (decreased expression) at FDR<0.05 by t-test for HNSCC samples only (SiHa and HCT116/54C were excluded).


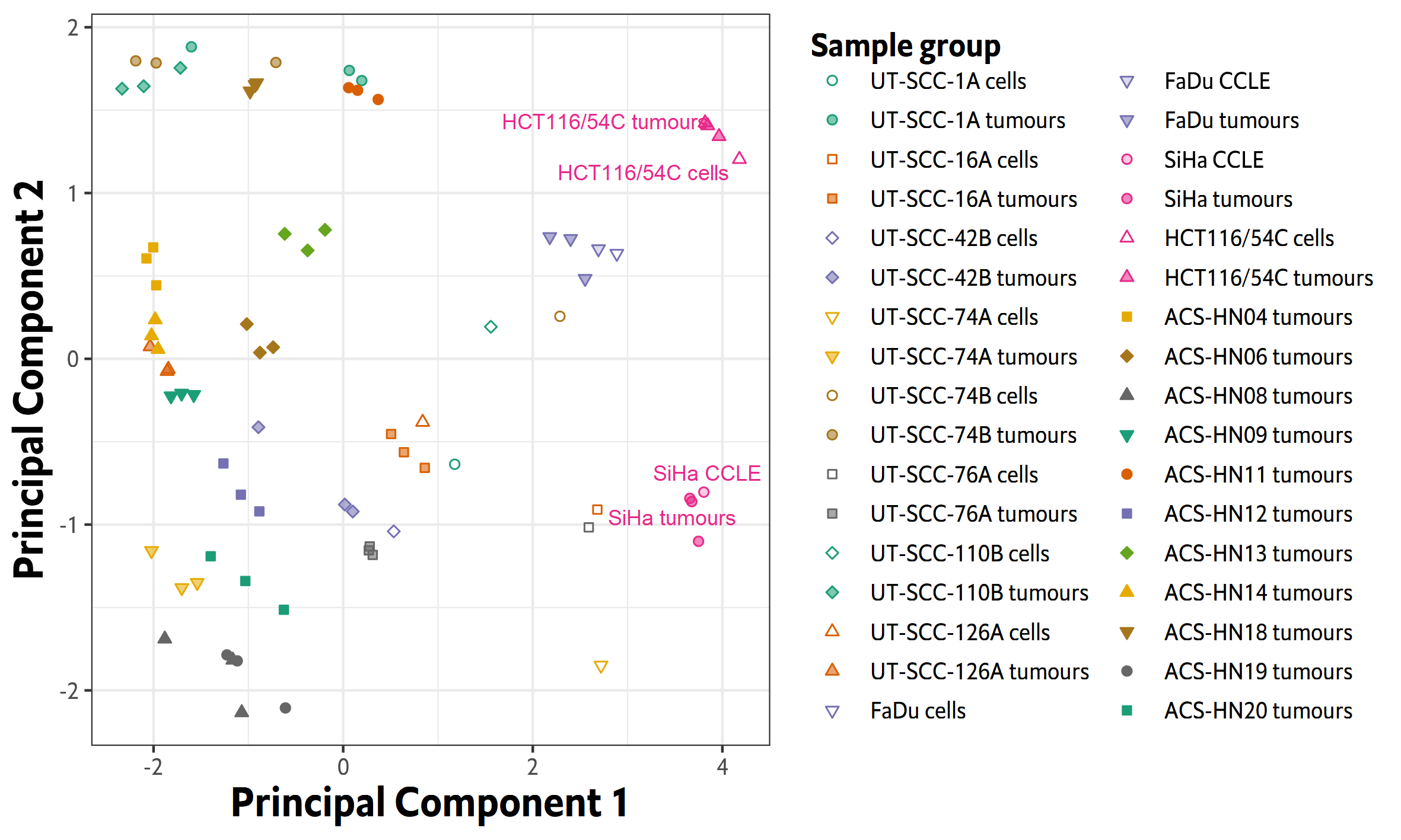


**Fig. S3** Principal component analysis for the log_2_ normalised counts per million of the 500 most variable genes for PDX and CDX tumours and cell lines. Non-HNSCC models are indicated.

**Buffa Signature**


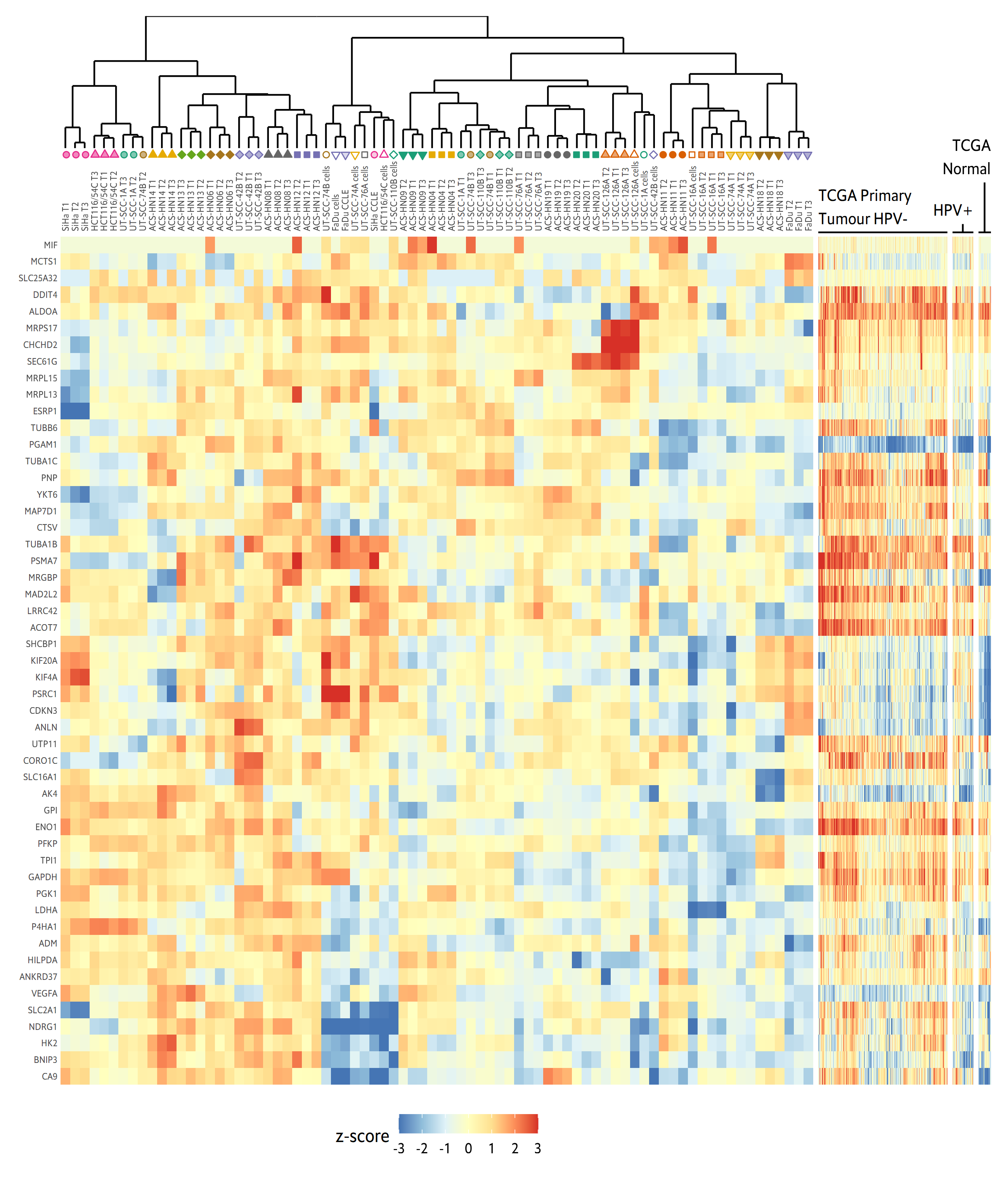


**Winter signature**


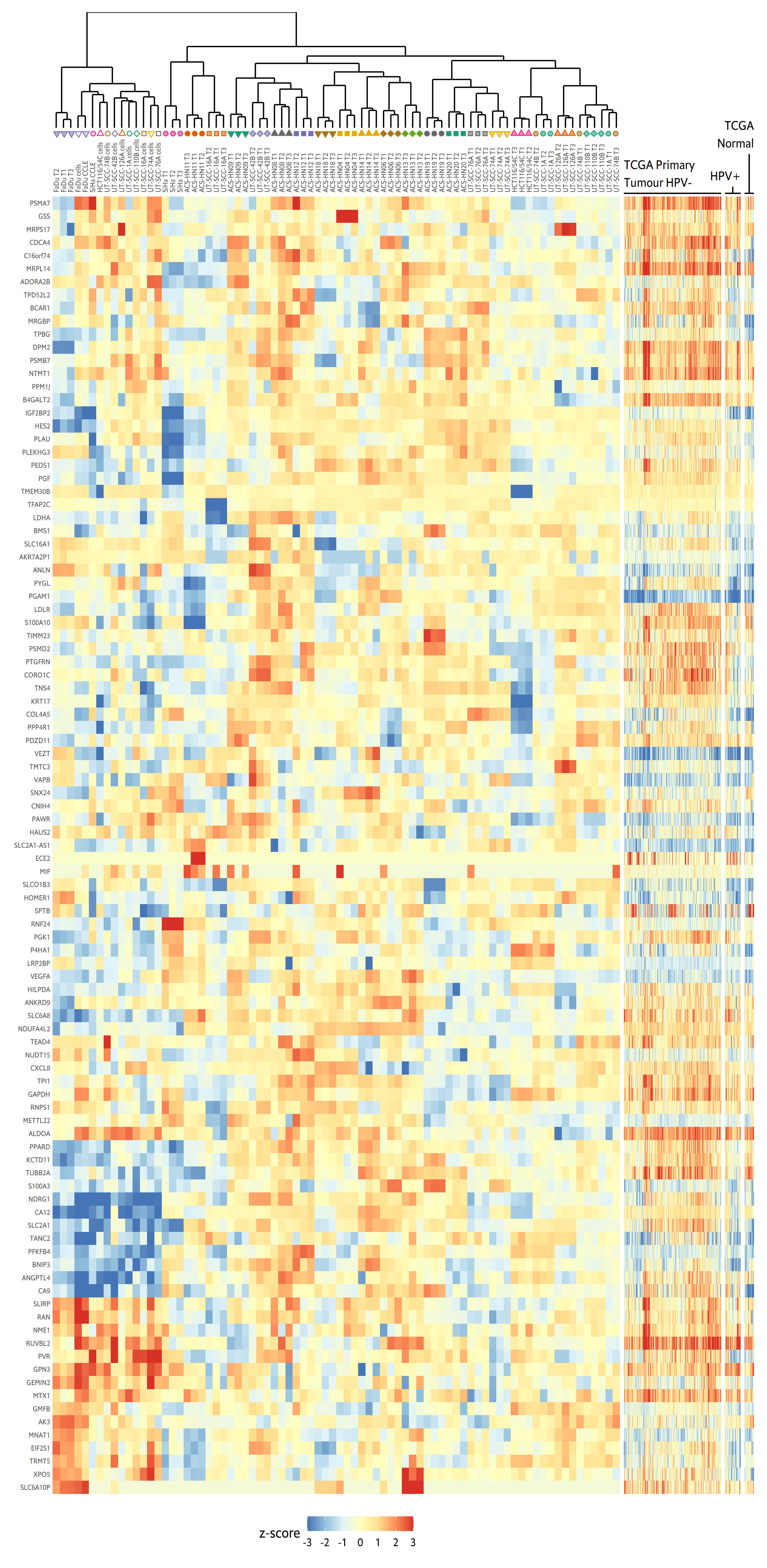
**Ragnum signature**


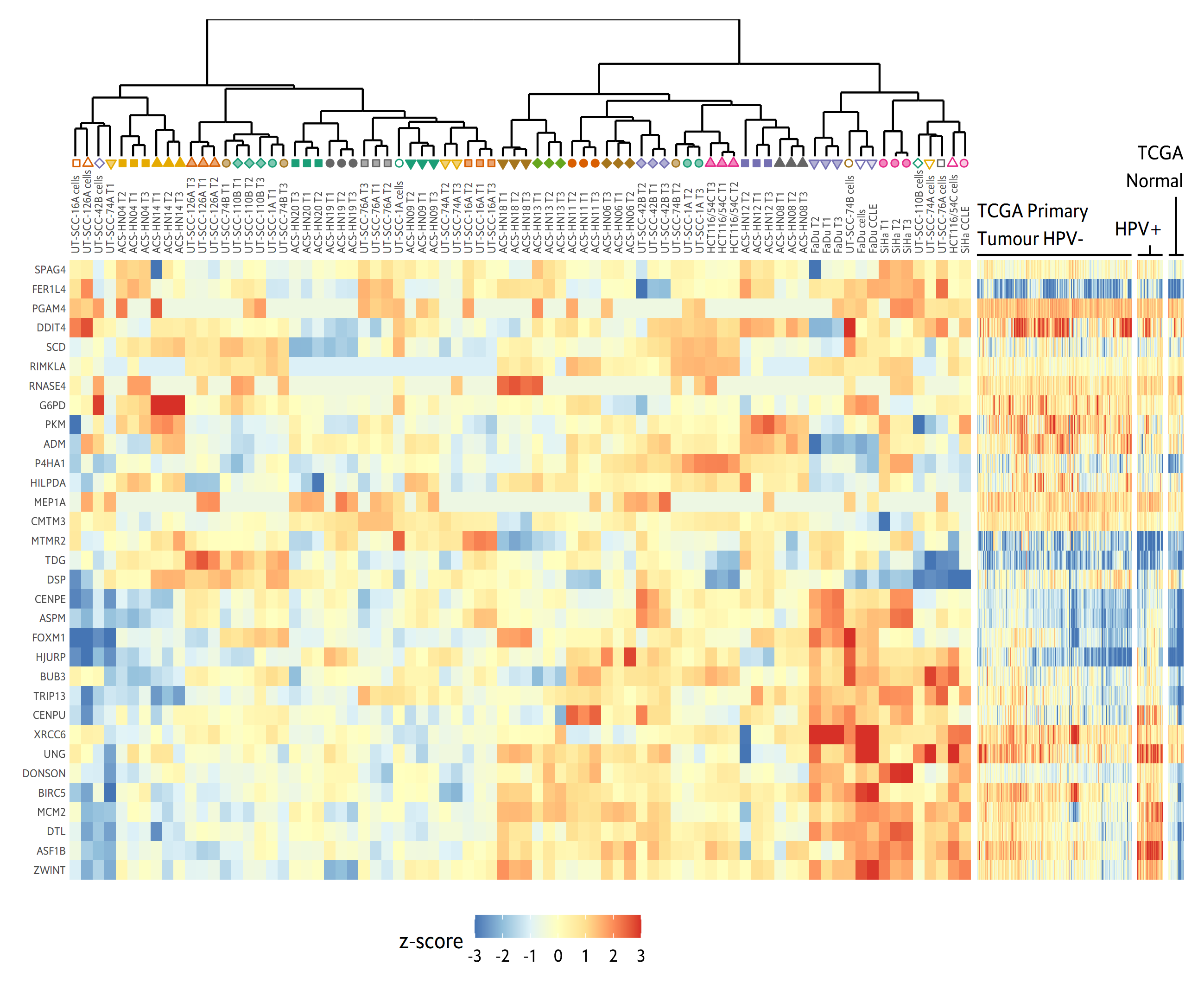


**Eustace signature
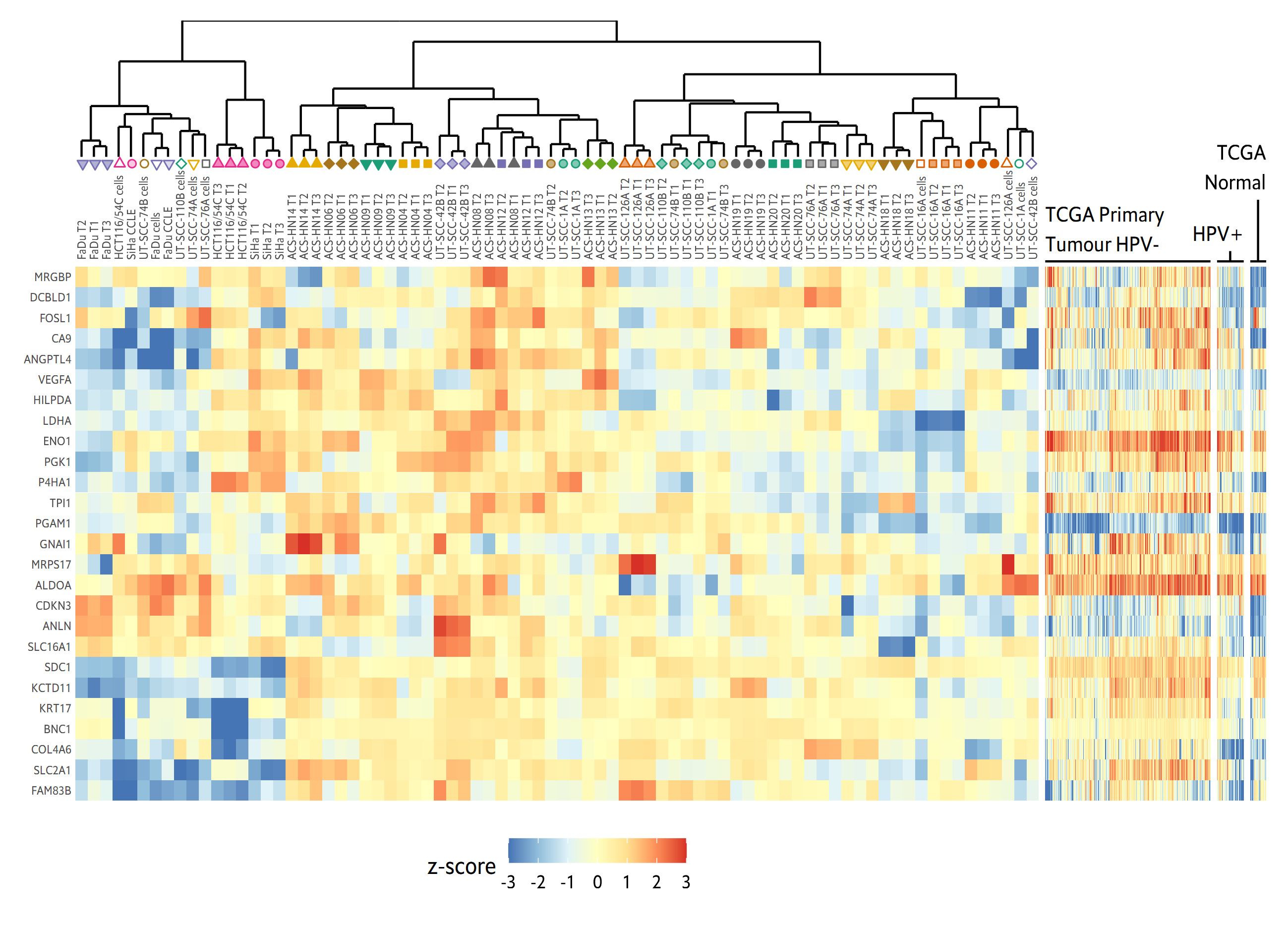
**

**Hu signature**

**
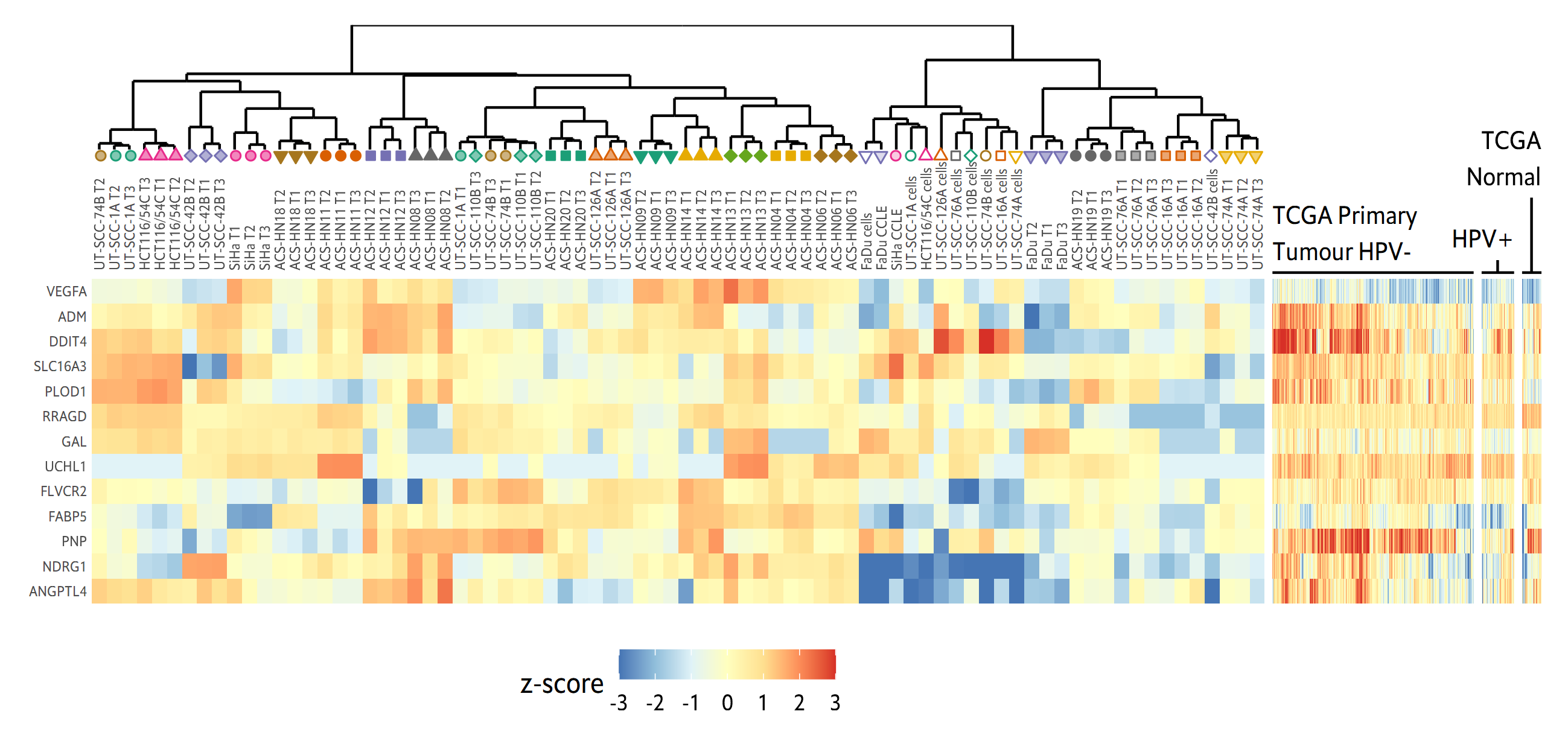
**

**Toustrup signature**

**
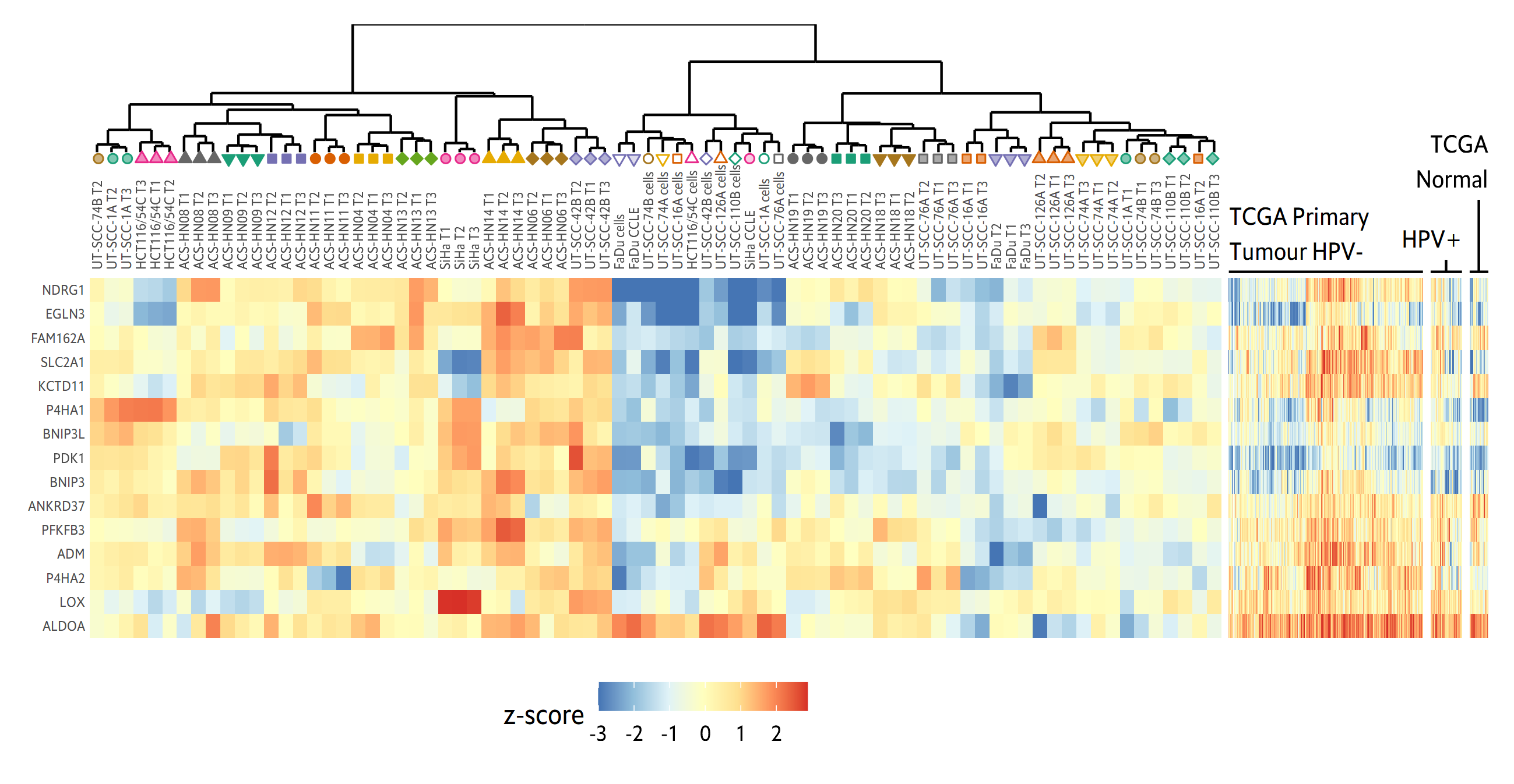
**

**Sung signature**

**
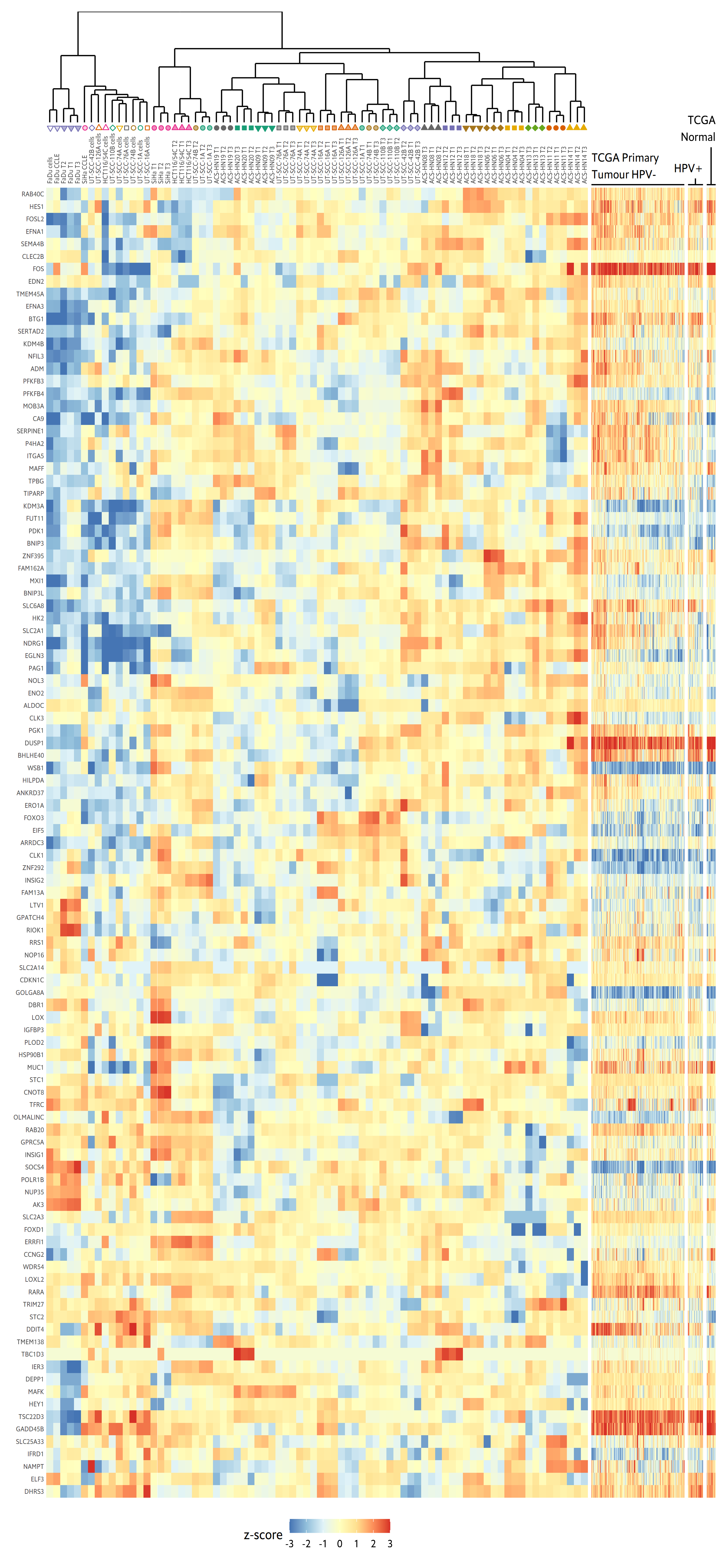
**

**Suh signature**

**
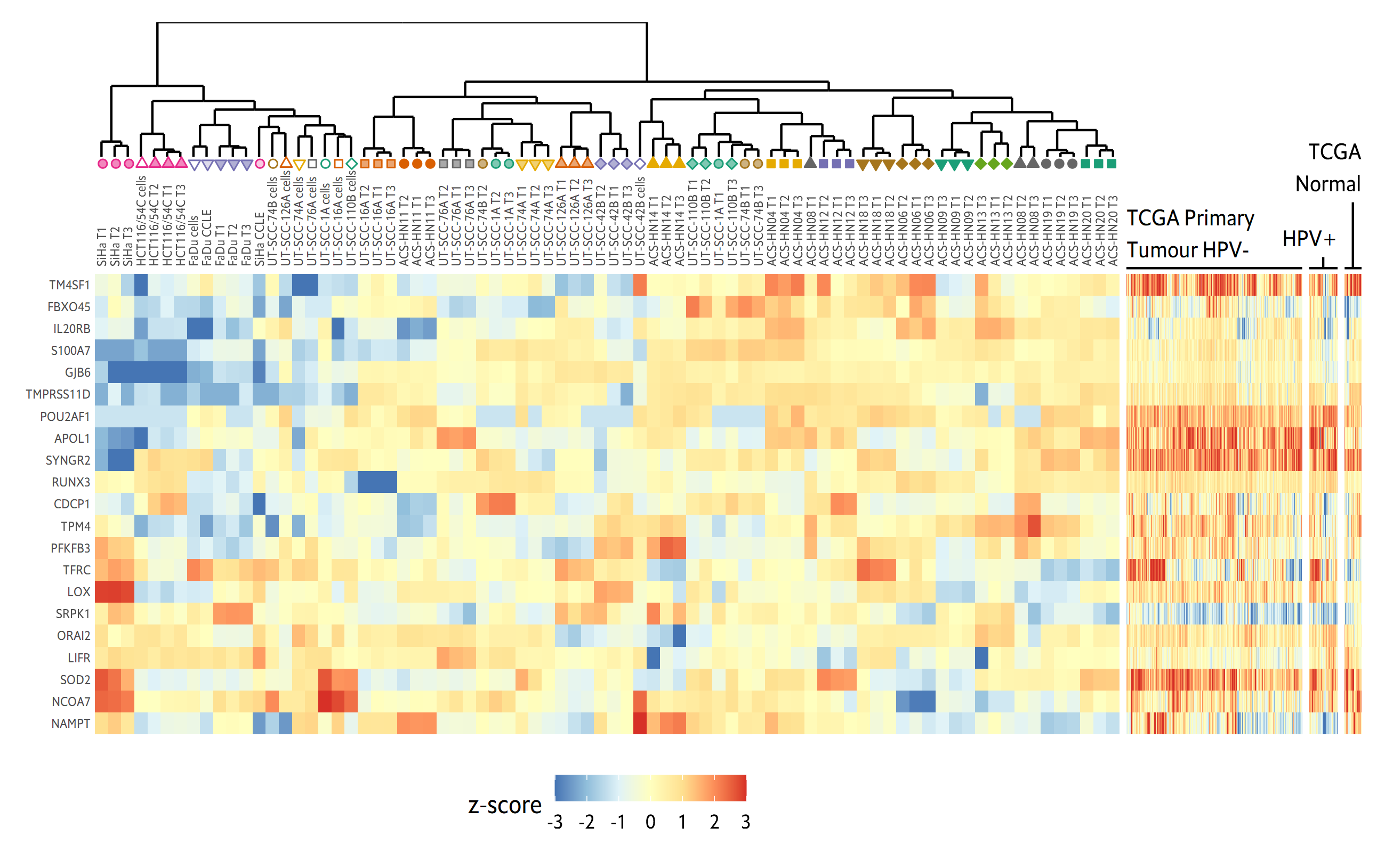
**

**Koong signature**

**
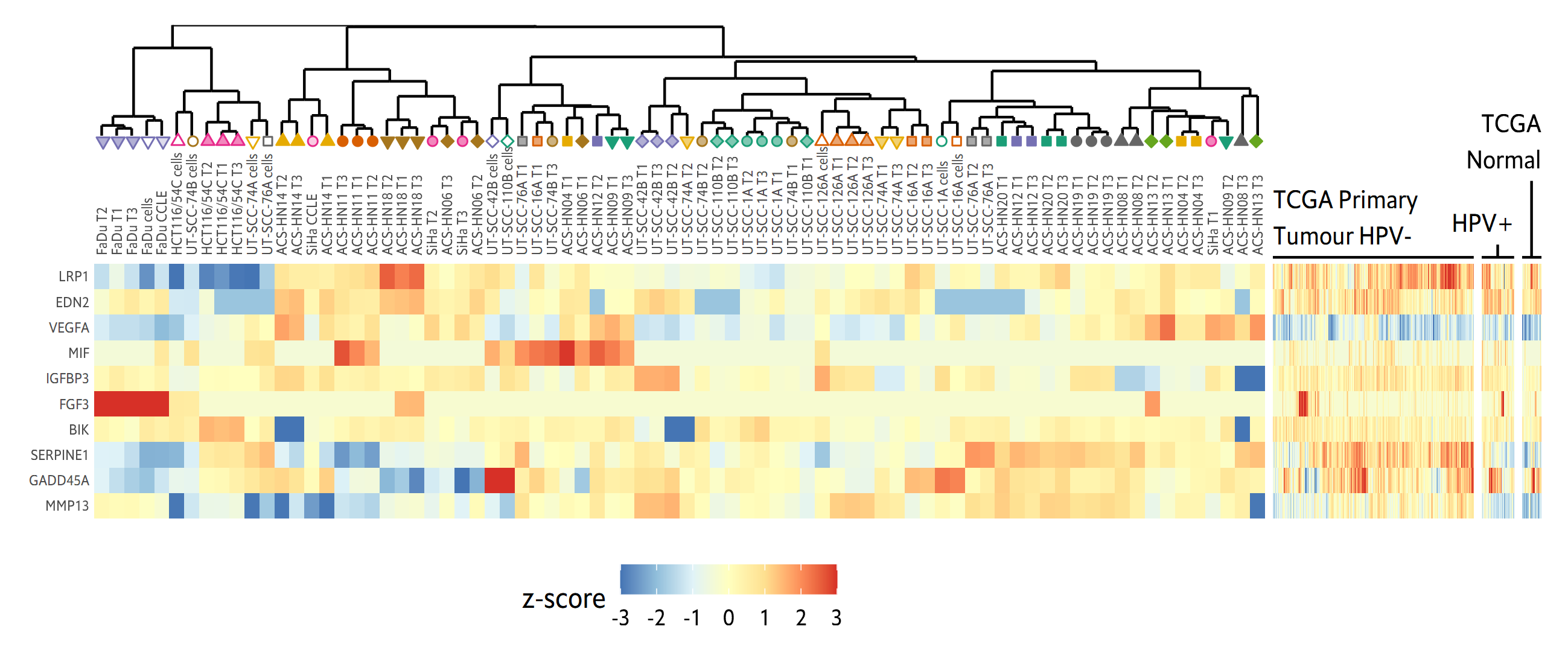
****Fig. S4** Heatmaps of gene expression for the genes used in the nine hypoxia gene signatures. Z-scores were calculated using mean and standard deviation of tumour xenograft samples and limited to the range of -3 to +3. TCGA Primary Tumour samples were categorised by HPV status prior to plotting. Samples within each subset and genes were hierarchically clustered by Euclidean distance using the Ward D2 method based on Z-score.

**Fig. S5** Signature scores for the nine hypoxia gene signatures for individual CDX and PDX tumours (n=3).

**Fig. S6** Comparison of Signature scores for the nine hypoxia gene signatures for paired CDX tumours and cell lines. Datapoints represent the average of three CDX tumours and individual cell cultures. *, P<0.05; ***, P<0.001 by paired T-test. Lines indicate the median sample

**Fig. S7** Hypoxia Scores for the non-HNSCC CDX tumours. Lines and error bars indicate the mean and standard error of the mean across the nine hypoxia signatures. Datapoints represent the average of three CDX tumours.

**Fig. S8** Comparison of Hypoxia Score and hypoxic fraction for HNSCC tumours for the nine hypoxia gene signatures. R values determined by Pearson correlation. Symbols and error bars represent mean ± SEM (n=3-5).

**Fig. S9** Comparison of Hypoxia Score and EdU count for HNSCC tumours for the nine hypoxia gene signatures. R values determined by Pearson correlation. Symbols and error bars represent mean ± SEM (n=3-5).

**Fig. S10** Tumour growth curves in mice with PDX or CDX tumours treated with 50 mg/kg evofosfamide in saline or control vehicle by IP injection at qd×5 for 3 weeks. Symbols represent mean and standard error for 6-11 animals per group. ACS-HN04 – ACS-HN14 are presented in [11].

a

b

c

**Fig. S11** Comparison of evofosfamide daily growth rate with a) Hypoxia Score, b) hypoxic fraction and c) EdU count for HNSCC tumour models. R values determined by Pearson correlation. Symbols and error bars represent mean ± SEM for 5-12 (x-axes) and 3-5 (y-axes) individual tumours for 18 tumour models in a), 16 tumour models in b) and 11 tumour models in c).
